# Mucosal Immunization with an Influenza Vector Carrying SARS-CoV-2 N Protein Protects Naïve Mice and Prevents Disease Enhancement in Seropositive Th2-Prone Mice

**DOI:** 10.3390/vaccines13010015

**Published:** 2024-12-28

**Authors:** Mariia V. Sergeeva, Kirill Vasilev, Ekaterina Romanovskaya-Romanko, Nikita Yolshin, Anastasia Pulkina, Daria Shamakova, Anna-Polina Shurygina, Arman Muzhikyan, Dmitry Lioznov, Marina Stukova

**Affiliations:** Smorodintsev Research Institute of Influenza of the Ministry of Health of the Russian Federation, 197022 St. Petersburg, Russia; kirill.vasilev@mssm.edu (K.V.);

**Keywords:** influenza vector, mucosal vaccine, SARS-CoV-2, inactivated vaccine, VAERD

## Abstract

**Background/Objectives:** Intranasal vaccination enhances protection against respiratory viruses by providing stimuli to the immune system at the primary site of infection, promoting a balanced and effective response. Influenza vectors with truncated NS1 are a promising vaccine approach that ensures a pronounced local CD8+ T-cellular immune response. Here, we describe the protective and immunomodulating properties of an influenza vector FluVec-N carrying the C-terminal fragment of the SARS-CoV-2 nucleoprotein within a truncated NS1 open reading frame. **Methods**: We generated several FluVec-N recombinant vectors by reverse genetics and confirmed the vector’s genetic stability, antigen expression in vitro, attenuation, and immunogenicity in a mouse model. We tested the protective potential of FluVec-N intranasal immunization in naïve mice and seropositive Th2-prone mice, primed with aluminium-adjuvanted inactivated SARS-CoV-2. Immune response in immunized and challenged mice was analyzed through serological methods and flow cytometry. **Results**: Double intranasal immunization of naïve mice with FluVec-N reduced weight loss and viral load in the lungs following infection with the SARS-CoV-2 beta variant. Mice primed with alum-adjuvanted inactivated coronavirus experienced substantial early weight loss and eosinophilia in the lungs during infection, demonstrating signs of enhanced disease. A single intranasal boost immunization with FluVec-N prevented the disease enhancement in primed mice by modulating the local immune response. Protection was associated with the formation of specific IgA and the early activation of virus-specific effector and resident CD8+ lymphocytes in mouse lungs. **Conclusions**: Our study supports the potential of immunization with influenza vector vaccines to prevent respiratory diseases and associated immunopathology.

## 1. Introduction

The continuous spread of COVID-19 since spring 2020, as well as the large-scale vaccination campaigns, has resulted in a high number of people becoming seropositive for SARS-CoV-2. By 2023, more than 5.55 billion people worldwide had received at least one dose of a coronavirus vaccine [1]. The reported worldwide seroprevalence rates averaged 59.2% before the appearance of the omicron strain [2]. Seroprevalence differed significantly depending on the country and specific population [3,4] and in some regions (e.g., Europe), reached 95.9% in March 2022 [2]. Therefore, studies on “prime–boost” vaccination—when a new vaccine is administered to individuals with pre-existing immunity to coronavirus—have become increasingly relevant.

As part of the Global COVID-19 Vaccine Strategy, the World Health Organization notes the need to invest in research and development of vaccines that are more effective and easier to administer, such as intranasal sprays and vaccines preventing transmission of the virus or providing wider and longer protection [5]. Influenza vector represents a promising candidate for the development of mucosal vaccines against respiratory pathogens targeting the nasal epithelia as the immunization site. Influenza vector attenuated by truncation of the NS1 protein ensures a prominent local T-cellular immune response to a heterologous insert, mediated by antigen-specific effector and central memory CD4+ and CD8+ T-lymphocytes [6]. The wide range of influenza antigenic subtypes enables multiple immunizations covering different targets. To date, several COVID-19 vaccine candidates based on influenza vectors have been described, with one of them already completing phase III clinical trials [ChiCTR2100051391].

The present study aims to develop and preclinically evaluate the FluVec-N attenuated influenza vector, which carries a SARS-CoV-2 N protein fragment in the truncated NS1 open reading frame. FluVec-N protective properties were assessed in naïve animals and in Th2-prone mice primed with an aluminum adjuvanted inactivated SARS-CoV-2 vaccine. The underlying mechanisms of vaccine-induced immune response including response in animals with pre-existing immunity upon subsequent SARS-CoV-2 infection are also discussed.

## 2. Materials and Methods

### 2.1. Recombinant Influenza Viruses

Recombinant influenza viruses were generated by reverse genetics [7]. Plasmids encoding the A/PR/8/34 (H1N1) virus gene segments were obtained from the collection of the Smorodintsev Research Institute of Influenza. Plasmids encoding the HA and NA genes of the A/Guangdong-Maonan/SWL1536/2019 (H1N1pdm09) virus were kindly provided by Prof. L. Rudenko (Institute of Experimental Medicine, St. Petersburg, Russia). To design the virus with a truncated NS1 (aa 1–124), the corresponding ORF in the pHW-PR8-NS plasmid was interrupted by inserting a multiple cloning site and a stop codon using site-directed mutagenesis. To generate the pHW-PR8-NS1_124__N plasmid carrying the fragment of the SARS-CoV-2 N protein (aa 211–369) fused to the NS1_124_ ORF, the corresponding nucleotide sequence was cloned from the hCoV-19/Russia/SPE-RII-3524V/2020 isolate into the multiple cloning site of the pHW-PR8-NS1_124_ plasmid.

Virus rescue was performed in serum-free cultivated Vero cells (ATCC #CCL-81, Manassas, VA, USA). Transfection of cells with plasmids (1 μg for each gene segment, 8 μg total per 3 × 10^6^ cells) was carried out through electroporation using Nucleofector kit V reagents (Lonza, Basel, Switzerland, #VCA-1003, EU) and the Nucleofector system (Lonza, EU). In 72 h post-transfection, rescued viruses were detected via hemagglutination and the cytopathic effect. Several subsequent virus passages were performed in 10-day-old chicken embryos (CEs) (JSC “Poultry Farm Sinyavinskaya”, St. Petersburg, Russia) at 34 °C with 48 h incubation.

The stability of the chimeric NS gene upon passaging was controlled through RT-PCR with primers specific to the influenza A NS gene UTR regions. The nucleotide sequence of viruses was verified via the whole-genome NGS sequencing.

### 2.2. SARS-CoV-2 Virus Isolation, Propagation, and Inactivation

Two SARS-CoV-2 viruses were used in the study: the B.1 lineage strain hCoV-19/Russia/StPetersburg-3524/2020 (GISAID EPI_ISL_415710) and the B.1.351 lineage strain hCoV-19/Russia/SPE-RII-27029V/2021 (GISAID EPI_ISL_1257814, beta variant). Viruses were isolated from human swab material and cultivated in Vero cells. Viral infectivity was measured using the standard TCID50 method, with detection based on cytopathic effect. Manipulations with live virus were carried out in a BSL3 facility.

SARS-CoV-2 was inactivated with 0.1% formalin (Vekton, St. Petersburg, Russia) at 37 °C for 72 h. The inactivated virus was purified via ultra-speed centrifugation through 30% sucrose cushion in the SW28 rotor (Beckman, Indianapolis, IN, USA) at 100,000× *g* for 3 h. The pelleted virus was resuspended in an NTE buffer, and total protein concentration was then measured on the Qubit fluorometer (Invitrogen, Carlsbad, CA, USA) using the Quant-iT Protein Assay. Virus inactivation was validated through sequential blind passages of inactivated material in Vero cells. Inactivation was confirmed if no cytopathic effect was observed on day 6 after the third blind passage.

### 2.3. Analysis of Antigen Expression in Infected Cells

Expression of proteins was analyzed at 6 h after infection of MDCK cells (IRR, #FR-58). Cells were grown in 6-well plates, then infected with 10 TCID_50_/cell of viruses and incubated at 37 °C, 5% CO_2_. Then cells were detached with Trypsin–EDTA (Sigma, St. Louis, MO, USA), pelleted via centrifugation, and lysed in 80 μL of 1× Laemmli buffer (Bio-Rad, Hercules, CA, USA) by heating at 100 °C for 7 min. Samples (5 μL) were loaded on the Mini-PROTEAN TGX Precast Gel (Bio-Rad, USA) and separated by SDS-PAGE. Then, protein bands were semi-dry transferred to nitrocellulose membrane (Bio-Rad, USA), and immunostained with 1H7 monoclonal antibody specific to influenza NS1 protein [8].

Vero cells in 96-well plates were infected with 10 TCID_50_/cell of recombinant viruses or the control SARS-CoV-2 virus (B.1 lineage) and incubated at 37 °C, 5% CO_2_. After 24 h, the cells were washed with DPBS (Biolot, St. Petersburg, Russia), fixed with 80% acetone (Vekton, Russia) in DPBS, and probed with FITC-labeled anti-influenza NP monoclonal antibody (LLC “PPDP”, St. Petersburg, Russia) or SARS-CoV-2 N protein-specific monoclonal antibody (Xema, Moscow, Russia) and AlexaFluor488-conjugated anti-mouse IgG antibody (ab150113, Abcam, Waltham, MA, USA). Images were captured using the Axio Vert.A1 fluorescent microscope with AxioCam ICc5 (Zeiss, Baltimore, MD, USA).

### 2.4. Laboratory Mice

Female C57/black and BALB/c mice (6–8 weeks old) were obtained from the Nursery for laboratory animals of the Shemyakin–Ovchinnikov Institute of Bioorganic Chemistry RAS (Pushchino, Russia). All animal studies were conducted in accordance with the Directive 2010/63/EU on the protection of animals used for scientific purpose and the protocols approved by the Bioethics Committee of the Smorodintsev Research Institute of Influenza (#44/1 dated 3 October 2021).

### 2.5. Safety and Immunogenicity Study

Naïve C57 mice were immunized intranasally with 10^6^ EID50 of the recombinant FluVec-N (H1N1) virus, the control Flu/NS124 (H1N1) vector without the insert, the wild type A/PR/8/34 (H1N1) virus, or DPBS in 30 μL under light ether anesthesia. Mice were weighed daily and monitored for lethality. On day 10 after immunization, 4 mice per group were euthanized and their lungs were collected to assess the cellular immune response to the influenza NP_366-374_ peptide (Verta, St. Petersburg, Russia) or the SARS-CoV-2 N-protein (a kind gift from the Saint Petersburg Scientific Research Institute of Vaccines and Serums of the FMBA of Russia).

### 2.6. Protection Study

Naïve BALB/c mice were immunized intranasally twice with 10^6^ EID50 of the recombinant FluVec-N vectors or the control empty vector in 30 μL under light ether anesthesia. The first immunization was performed with the H1N1pdm09 virus, and the second with the H1N1 virus. For prime–boost immunization, mice were first injected intramuscularly with 2.5 μg of formalin-inactivated SARS-CoV-2 adsorbed to 10 μg aluminum hydroxide adjuvant (Sigma, USA) and then immunized intranasally with the FluVec-N (H1N1) virus. Mice that received only the prime immunization served as the control group. The placebo control group was given DPBS intranasally twice. Intact mice were used as background control in immunological experiments. Each experimental and control group included 15 mice, except for the intact group which included 5 mice. Five mice in each group (except intact) were euthanized two weeks after the second immunization to analyze neutralizing SARS-CoV-2 and N-specific antibodies in serum and broncho-alveolar lavage (BAL), respectively.

Three weeks after the second immunization, 10 mice in each group (except intact) were intranasally infected with 10^5^ TCID50 of the SARS-CoV-2 beta variant in 50 μL volume under light ether anesthesia. Mouse weight was measured daily after infection. On day 5 post-infection, 5 mice in each group and 2 intact mice were euthanized to analyze viral load in the nasal turbinates and BAL and to estimate innate and adaptive cellular immune response in the lungs. On day 7 after infection, the last 5 mice in each group and 3 intact mice were euthanized to perform histopathological evaluation of the lungs.

### 2.7. Antibody Assessment

Neutralizing antibodies to the B.1 SARS-CoV-2 virus were assessed using a classical TCID50-based microneutralization assay. Briefly, sera were heat-inactivated at 56 °C for 1 h, prediluted 1:10 in culture medium, then subjected to 2-fold serial dilution in U-well plates (Medpolimer, St. Petersburg, Russia) and mixed with an equal volume of infectious virus containing 100 TCID50/well. Mixtures were incubated at 37 °C for 1 h and then transferred to Vero cells in 96-well plates (seeded with density 2 × 10^6^ cells/well) and incubated at 37 °C, 5% CO_2_ for 4–5 days. The plates were read manually under a light microscope, and the neutralizing titer was calculated as the last serum dilution that inhibited cytopathic effect formation.

Secretory IgA in mouse BALs was assessed through ELISA. Maxisorp plates (Nunc, Roskilde, Denmark) were covered with 200 ng/well of recombinant N-protein and incubated at 2–8 °C overnight. Next, plates were washed two times with 500 μL/well of 0.1% Tween20 (Amresco, Solon, OH, USA) in PBS (Biolot, St. Petersburg, Russia) and blocked with 100 μL/well of 5% skimmed milk (Stoing, St. Petersburg, Russia) in wash solution at room temperature for 2 h. BALs were diluted 1:2 in blocking solution, and 100 μL samples were added to plate wells and incubated at room temperature for 1.5 h. Then plates were washed four times and incubated with 100 μL/well of HRP-labelled secondary antibody (Anti-Mouse IgA (a-chain) Peroxidase Goat Ab, A4789, Sigma, USA) diluted 1:1000 in blocking solution. After 1 h of incubation, plates were washed six times and filled with 100 μL/well of ready-to-use TMB substrate solution (Bioservice, Novgorod, Russia). The colorimetric reaction was allowed to proceed for 15 min in the dark and then stopped by adding 100 μL of 1M H2SO4 (Vekton, St. Petersburg, Russia) per well. Optical density was measured at a wavelength of 450 nm using the CLARIOstar microphotometer (BMG LABTECH, Cary, NC, USA), with background subtraction at 655 nm.

### 2.8. Histopathological Analyses

Lung tissue was fixed in a 10% neutral buffered formalin solution, dehydrated in increasing concentrations of isopropanol, embedded in paraffin, sectioned, and stained with hematoxylin and eosin. Histological examination was performed using the Axioscope 2 Plus light microscope (Zeiss, Jena, Germany), and microphotographs were taken with the AxioCam ERc5s digital camera and the AxioVision Rel software 4.8 (Zeiss, Germany). Pathological changes were estimated using the conventional 5-point scale. Tissue damage (0–5), acute inflammation (0–5), and lymphocyte infiltration (0–5) were evaluated in the following respiratory tract compartments: bronchioli, blood vessels, interstitium, and alveoli. Scores for all compartments were summarized to calculate a single summary score for each animal.

### 2.9. Cellular Immune Response

To study the activation of the innate and adaptive cellular immune response, mice were sacrificed via cervical dislocation, the left ventricle was perfused with 10 mL of cold DPBS, and then the lungs were removed. Organs were homogenized using the Tissue Lyser II homogenizer (Qiagen, Hilden, Germany) and treated with a mixture of collagenase (Sigma) and DNase I (Sigma) at 37 °C for 30 min. Tissue homogenates were passed through a 70 μm cell filter. Cells were washed once (500 g, 7 min) in DPBS containing 2.5% FBS (Gibco, Grand Island, NY, USA). RBC lysis was performed using the RBC Lysis Buffer reagent (Biolegend, San Diego, CA, USA). Cells were washed again in DPBS + 2.5% FBS. For further analysis, 2 × 10^6^ cells were selected. Cell counts were evaluated using a Cytoflex flow cytometer (Beckman Coulter, Brea, CA, USA).

#### 2.9.1. Assessment of the Innate Immune Response

**Figure A1:**
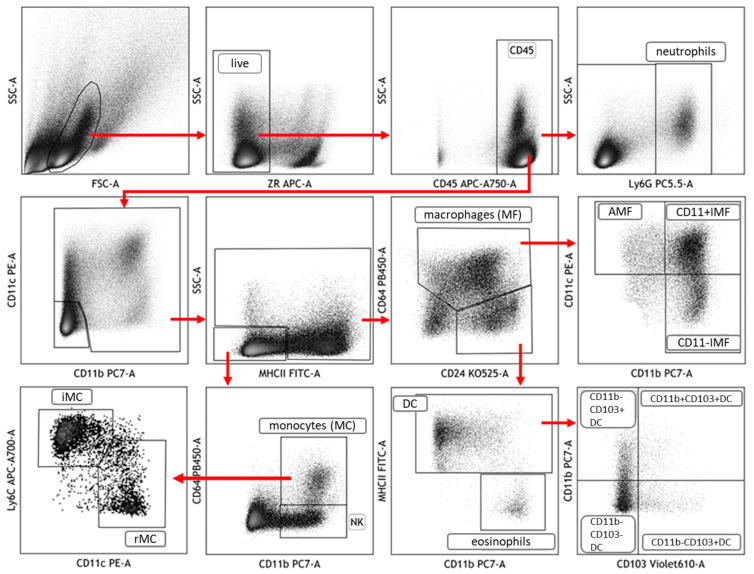
Gating strategy to identify innate immune cell populations.

To study the cellular composition of the innate immunity, a panel of fluorescently labeled antibodies was used: CD11b-PE/Cy7, CD11c-PE, MHCII-Alexa488, Ly6G-PerCP-Cy5.5, Ly6C-Alexa700, CD103-BV610, CD45-APC/Cy7, CD64-BV421, CD24-BV510 (Biolegend, USA). Cell staining was carried out in V-bottom 96-well plates. Data acquisition was performed with a Cytoflex flow cytometer and the results were analyzed using Kaluza Analysis 2.1 (Beckman Coulter, Brea, CA, USA). The gating strategy for identifying particular cell populations is presented in Appendix A Figure A1. The panel used allows identifying populations of macrophages (alveolar macrophages (AMP), interstitial macrophages CD11c+/CD11c−), monocytes (inflammatory monocytes, resident monocytes), dendritic cells (CD11b+CD103+, CD11b-CD103+, CD11b+CD103−, CD11b-CD103−), NK cells, eosinophils, and T and B lymphocytes.

#### 2.9.2. Assessment of the Adaptive Immune Response

**Figure A2:**
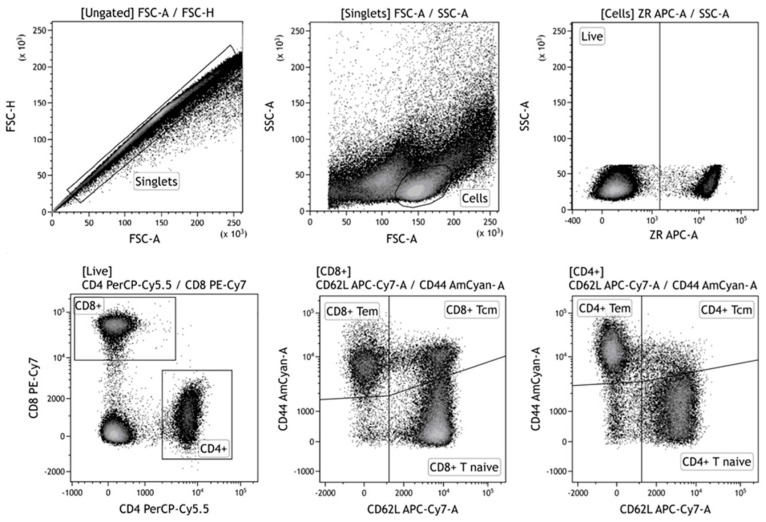
Gating strategy to identify adaptive immunity cell populations.

To assess the adaptive immune response, lung lymphocyte cells were washed in RPMI medium containing 10% fetal calf serum and 1% mixture of penicillin and streptomycin (Gibco), scattered into flat-bottomed plates with a density of 2 × 10^6^ cells/100 µL, and stimulated with a cocktail of SARS-CoV-2 virus N protein peptides (PepTivator^®^ SARS-CoV-2 Prot_N, Miltenyi Biotec, Santa Barbara, CA, USA). The cell transport inhibitor brefeldin A (BD Biosciences, Franklin Lakes, NJ, USA) was added to the medium. Stimulation was carried out for 6 h in the presence of costimulatory antibodies to CD28 (Biolegend). Background control wells contained the same reagents, except for the peptide pool. After stimulation, cells were stained with fluorochrome-conjugated antibodies CD8-PE/Cy7, CD4-PerCP-Cy5.5, CD44-BV510, CD62L-APC/Cy7, IFNγ-FITC, TNFα-BV421, IL2-PE (Biolgend), and Zombie Red (Biolegend) to measure cell viability. Nonspecific antibody binding was prevented by using the True Stain reagent containing antibodies to CD16/CD32 (Biolegend). Staining was performed using the Cytofix/Cytoperm intracellular staining kit (BD Biosciences) according to the manufacturer’s instructions. The gating strategy for identifying particular cell populations is presented in Appendix A Figure A2. Data acquisition was performed with a Cytoflex flow cytometer and results were analyzed using Kaluza Analysis v2.1.

### 2.10. Statistical Analyses

Statistical analysis and data visualization were performed using GraphPad Prism v8.4.3 and RStudio version 1.4.1106 software. Quantitative data were analyzed using the following descriptive statistics: mean and standard deviation (SD), geometric mean and SD for the geometric mean, and median (Me) with interquartile range (IQR). Statistical differences between groups were assessed using analysis of variance, followed by multiple pairwise comparisons with *p*-value correction.

## 3. Results

### 3.1. FluVec-N Recombinant Viruses Are Genetically Stable, Attenuated, and Immunogenic in Mice

**Figure 1:**
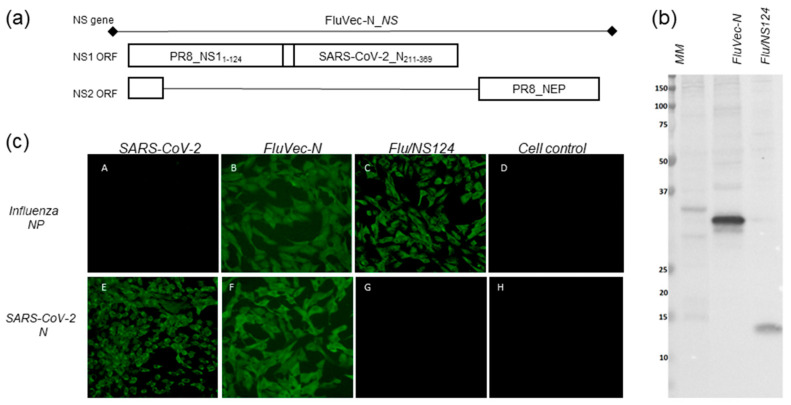
The NS gene structure of the recombinant FluVec-N virus and expression of the chimeric NS1_124__N protein. (**a**) Proteins encoded by two ORFs in the NS gene. (**b**) Western blot of infected cell lysates probed with anti-NS1 antibody. Viruses are indicated at the top; molecular weight marker is shown in kDa. (**c**) Immunofluorescent microscopy images of infected cells probed with anti-influenza NP antibody (A–D) and anti-SARS-CoV-2 N protein antibody (E–H). Viruses are indicated above the panel. The original images can be found in the Supplementary Materials.

Recombinant influenza viruses FluVec-N, carrying the SARS-CoV-2 N protein C-terminal fragment (aa 211–369) fused to the truncated NS1 (Figure 1a), were generated via reverse genetics in Vero cells and propagated in 10–12-day old CE. Two FluVec-N viruses were constructed on the backbone of the influenza A/PR/8/34 (H1N1) strain (PB2, PB1, PA, NP, M, and modified NS). FluVec-N (H1N1) virus inherited HA and NA from the A/PR/8/34 and FluVec-N (H1N1pdm) virus inherited HA and NA from A/Guangdong-Maonan/SWL1536/2019 (H1N1pdm09) virus. Empty Flu/NS124 vectors with the truncated NS1_1–124_ protein and the same surface antigens (H1N1 or H1N1pdm09), as well as the A/PR/8/34(H1N1) backbone virus with the wild type NS, were generated the same way entirely from plasmids.

**Figure A3:**
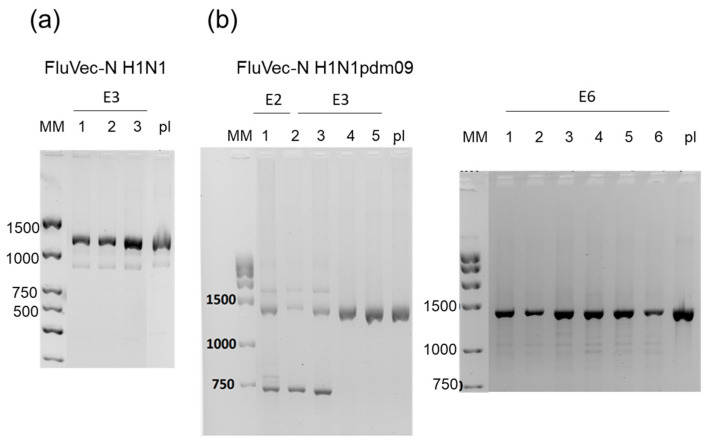
Genetic stability of the FluVec-N chimeric NS gene. The whole-length RT-PCR amplification products are presented; pl—gene amplicon from the control plasmid. (**a**) Length of the NS gene segment of the virus clones from the 3rd passage of the FluVec-N (H1N1) recombinant virus carrying HA and NA from the A/PR/8/34 (H1N1) strain; (**b**) Length of the NS gene segment of the virus clones from the 2nd, 3rd, and 6th passages of the recombinant FluVec-N (H1N1pdm09) virus carrying HA and NA from the A/Guangdong-Maonan/SWL1536/2019 (H1N1pdm09) strain. The original images can be found in Supplementary Materials.

Recombinant FluVec-N strains were genetically stable upon passaging in CE (Appendix B Figure A3) and characterized by infectious activity exceeding 8.5 lgEID50/mL. The expression of the truncated NS1 protein with the heterologous N protein insert by recombinant viruses in infected cells was confirmed through Western blot analyses (Figure 1b) and immunofluorescent staining (Figure 1c). The detected molecular weight of the NS1_124__N chimeric fusion protein corresponded to the theoretically predicted 32 kDa, and the NS1_124_ protein band was detected near the 14 kDa level, as predicted using the EXPASY ProtParam online tool.

**Figure 2:**
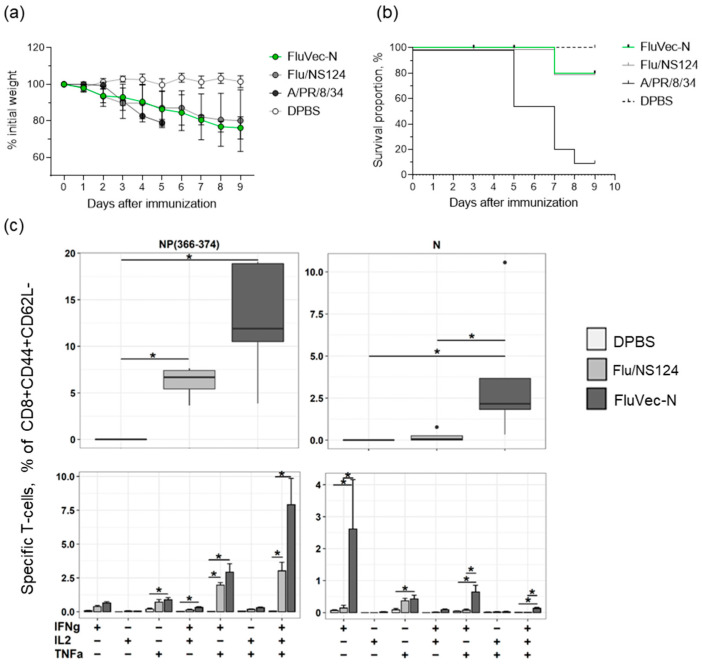
The recombinant FluVec-N virus is attenuated and immunogenic in C57 mice. (**a**) Weight dynamics of mice intranasally inoculated with the indicated viruses, shown as percent of the initial weight (M ± SD). (**b**) Survival of mice intranasally inoculated with the indicated viruses. (**c**) T-cell immune response in mouse lungs to the influenza NP (366–374) peptide (left) or the SARS-CoV-2 N-protein (right) 10 days after immunization with the indicated virus. Relative content of total (upper panel) and individual (lower panel) subpopulations of cytokine-producing effector CD8+ T lymphocytes. Data obtained after subtracting background values of the relative content of cytokine-producing cells in the unstimulated control are presented. Statistical analysis was performed using ANOVA (*p* < 0.0001), followed by pairwise group comparison using Tukey’s test. * *p* < 0.05 marks significant differences with the DPBS control group.

Attenuation and immunogenicity of the FluVec-N recombinant viruses were confirmed in C57 mice upon intranasal administration. Representative data (Figure 2a,b) shows weight dynamics and survival of mice inoculated with six lgEID50 of the FluVec-N (H1N1) virus and the empty Flu/NS124 virus vector in comparison to the wild type backbone A/PR/8/34 (H1N1) virus with the full-length NS1. Our results demonstrate that similar to the empty vector, the FluVec-N virus was safe in mice, unlike the lethal A/PR/8/34 (H1N1) virus.

**Figure A4:**
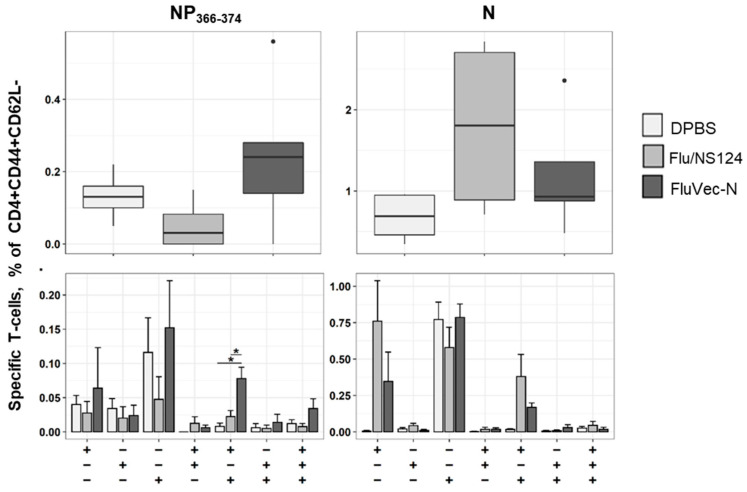
CD4+ T-cell response to the influenza NP (366–374) peptide and the SARS-CoV-2 N-protein in the lungs of immunized mice. Relative content of total (upper panel) and individual (lower panel) subpopulations of cytokine-producing effector CD4+ T lymphocytes was assessed 10 days after immunization with the indicated viruses. Data obtained after subtracting background values of the relative content of cytokine-producing cells in the unstimulated control are presented. Statistical analysis was performed using ANOVA (*p* < 0.0001) followed by pairwise group comparison using Tukey’s test. * *p* < 0.05 marks significant differences with the DPBS control group.

A single intranasal immunization with the recombinant FluVec-N virus induced the formation of a pronounced SARS-CoV-2 N-specific CD8+ T-cell response in mice (Figure 2c). The IFNγ+IL2-TNFα- population played the predominant role. A significant increase in the relative content of polyfunctional CD8+ T-lymphocytes (IFNγ+IL2-TNFα+ and IFNγ+IL2+TNFα+) was also noted. The antigen-specific CD8+ T-cell response to the influenza virus NP_366–374_ peptide epitope was mediated by the activation of polyfunctional cells: IFNγ+IL2-TNFα+ and IFNγ+IL2+TNFα+. At the same time, immunization with FluVec-N did not significantly affect the CD4+ N-specific T-cell response (Appendix C Figure A4).

### 3.2. FluVec-N Protecs Naïve and Primed Mice Against SARS-CoV-2 Infection

**Figure 3:**
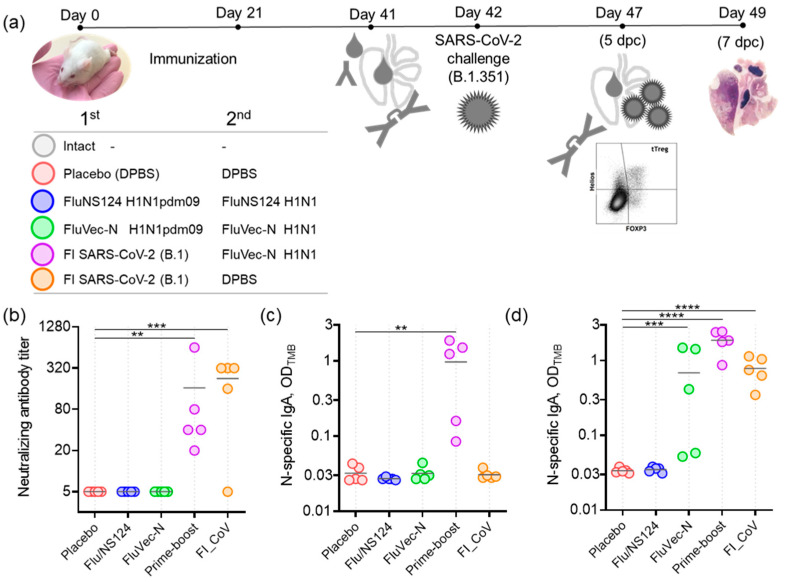
Protection experiments in BALB/c mice. (**a**) Study design. Immunization schemes for experimental groups are listed in the lower left, FI—formalin-inactivated. (**b**) Neutralizing SARS-CoV-2 antibodies in serum after second immunization. (**c**) N-protein specific antibodies in BAL after second immunization, sample dilution 1/2. (**d**) N-protein specific antibodies in BAL after challenge, sample dilution 1/2. ANOVA (*p* < 0.0001) was followed by Dunnette’s test for multiple comparisons of each group with placebo: ** *p* < 0.01, *** *p* < 0.001, **** *p* < 0.0001.

Immunogenic and protective properties of FluVec-N against SARS-CoV-2 were assessed in naïve and primed Th2-prone BALB/c mice. Naïve BALB/c mice received double immunization with recombinant FluVec-N vectors (first immunization with H1N1pdm09 vector, second with H1N1 vector). Priming was performed via preimmunization with alum-adjuvanted formalin-inactivated SARS-CoV-2, inducing Th2 immune response polarization [9]. Primed mice further received one booster immunization with the recombinant FluVec-N (H1N1) virus. Control groups included mice who received only the inactivated SARS-CoV-2 formulation, an empty vector, or a placebo (as the infection control group). Challenge with SARS-CoV-2 was performed three weeks after the last immunization (Figure 3a).

#### 3.2.1. FluVec-N Immunization Activates the Local Humoral Immune Response and Fast Antibody Recall in the Respiratory Tract upon Challenge

Pre-challenge immune response to SARS-CoV-2 was assessed two weeks after the last immunization. As expected, serum SARS-CoV-2 neutralizing antibodies were detected only in mice who received inactivated SARS-CoV-2 formulation as a single immunization or in the prime–boost regime, with no difference in geometric mean titers between these two groups (Figure 3b). N-specific local antibodies in BALs were detected only in the group that received prime–boost immunization (Figure 3c). Further, secretory antibodies were also assessed in BALs collected 5 days post-challenge (dpc; Figure 3d). Infection led to the early elevation of N-specific antibodies in mice immunized with the inactivated SARS-CoV-2 formulation or FluVec-N, but not in the empty vector and placebo groups. The obtained results demonstrate that FluVec-N immunization alone did not activate the formation of detectable levels of N-specific secretory antibodies in the respiratory tract, although it may produce memory cells that trigger fast antibody recall upon infection.

#### 3.2.2. FluVec-N Immunization Reduces Viral Load and Tissue Damage upon Infection

**Figure 4:**
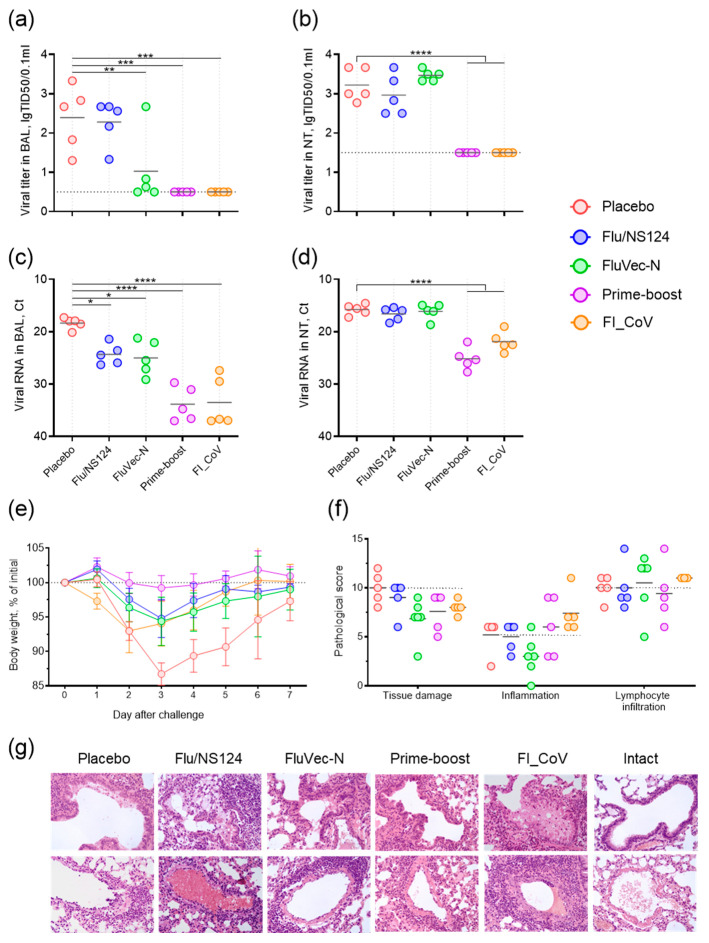
Protection experiments in BALB/c mice. (**a**,**b**) Infectious titers in BAL and the nasal turbinates (NTs) of challenged mice at 5 dpi. (**c**,**d**) Virus RNA in BAL and NTs of challenged mice at 5 dpi. Individual values and group means are presented. ANOVA (*p* < 0.0001) was followed by Dunnette’s test for multiple comparisons of each group with placebo: * *p* < 0.05, ** *p* < 0.01, *** *p* < 0.001, **** *p* < 0.0001. (**e**) Body weight of challenged mice during a week after infection. Group means with 95% confidence intervals are presented. (**f**) Histopathological summary score of lung examination at 7 dpi. Individual values and group means are presented. The dotted line corresponds to the mean value for the placebo (infection control) group. (**g**) Microphotographs of the most pronounced pathological changes in bronchioles (upper panel) and blood vessels (lower panel) in the mouse lungs at 7 dpi, 400×.

To model the COVID-19 disease, we used the SARS-CoV-2 beta variant that is antigenically distant from the B.1 lineage used for immunization and can infect BALB/c mice without prior adaptation [10,11]. Infection with the beta variant is characterized by more active replication of the virus in the nasal turbinates than in the lungs, and a drop in viral load starting two days post-infection (dpi). As the N-specific response is aimed at virus clearance rather than the prevention of the virus infection, we evaluated viral load in mouse lungs and nasal turbinates at a later time point, 5 dpi. Infected animals from the placebo group were found to have live virus in the lungs and nasal turbinates with the mean titers of 2.4 lgTID50 and 3.2 lgTID50, respectively. The empty vector control group showed a similar viral load (Figure 4a,b). Mice immunized twice with FluVec-N had reduced infectious virus in the lungs in comparison to the placebo group, with an average decrease of 1.4 lgTID50; viral titers in the nasal turbinates were the same. Mice vaccinated with the inactivated virus or in the –boost regime showed no presence of infectious virus in either the lungs or the nasal turbinates. Viral RNA was detected through real-time RT-PCR in all animals (Figure 4c,d), indicating that priming with the inactivated formulation did not provide sterilizing immunity. Moreover, animals immunized with the inactivated virus alone exhibited early weight loss, which reached 7% at 2 dpi and was comparable with the placebo group (Figure 4e). The peak weight loss was detected on day 3 for animals from the placebo group (13%). Animals immunized in the prime–boost regime did not show any signs of weight loss.

Histopathological analyses revealed a trend toward reduced tissue damage and acute inflammation in mice immunized with FluVec-N in comparison to placebo (Figure 4f,g), while mice vaccinated with the inactivated virus showed the most severe pathology. All infected animals in the placebo group had perivasculitis, whereas mice vaccinated with FluVec-N mostly maintained the normal histological structure of the lung vessel walls. In animals primed with the inactivated virus, focal inflammatory damage to the walls of blood vessels was observed as mild vasculitis and moderate perivascular inflammatory infiltrates. In animals vaccinated in the prime–boost scheme, perivascular lymphocytic infiltrates were observed and vasculitis was much less common and minimally expressed. In some animals from this group, the extent of pathology and the area of pulmonary tissue damage were much lower.

### 3.3. Immune Response upon SARS-CoV-2 Infection in Mice Immunized with FluVec-N

#### 3.3.1. Innate Immunity Patterns in the Lungs of Mice upon Subsequent Infection

**Figure 5:**
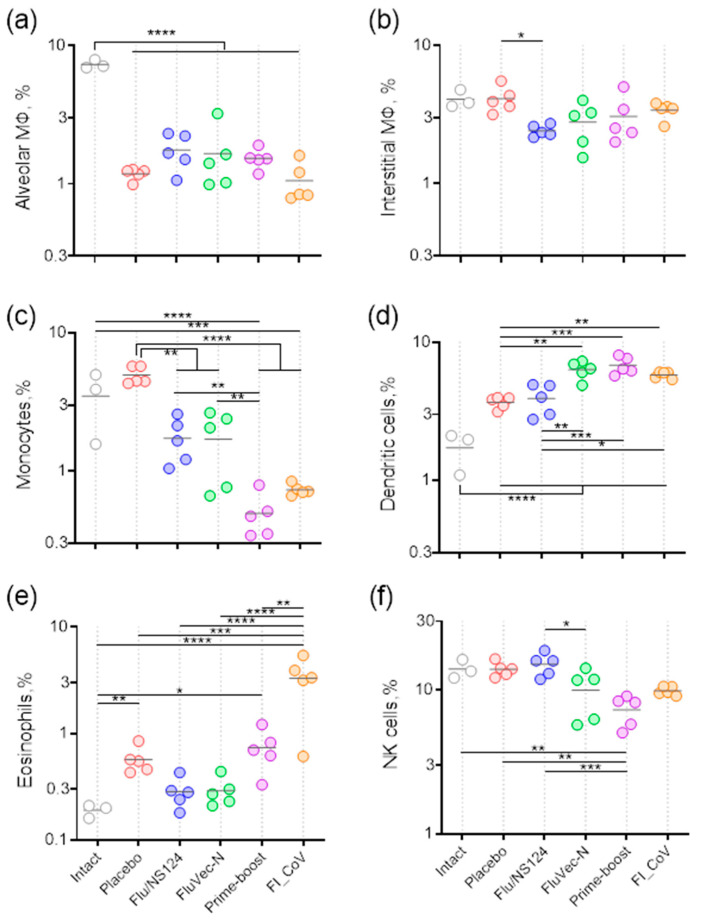
Innate immunity populations in the lungs of vaccinated BALB/c mice upon subsequent infection with SARS-CoV-2. (**a**) Alveolar macrophages; (**b**) interstitial macrophages; (**c**) monocytes; (**d**) dendritic cells; (**e**) eosinophils; (**f**) natural killers. Percentages of different cell types in the population of lung CD45+ cells are presented individually for each animal, and the horizontal line represents the group mean. An intact group is presented for comparison with normal non-infected mice. * *p* < 0.05, ** *p* < 0.01, *** *p* < 0.001, **** *p* < 0.0001 calculated using Tukey post-hoc test following one-way ANOVA applied to log values (*p* < 0.0001).

On day five after SARS-CoV-2 infection, the relative content of the main populations of innate immunity cells in the lungs of mice was analyzed through flow cytometry. All infected animals, regardless of the vaccination method, showed a significant decrease in the proportion of alveolar macrophages in the lung tissue alongside an increase in dendritic cells compared to intact mice (Figure 5a,b,d). In the placebo group, as well as in mice who received the empty vector, the relative content of dendritic cells was lower than in mice vaccinated with FluVec-N. This can be explained by differences in the composition of the cytokine microenvironment formed in the pulmonary interstitium of animals with or without antigen-specific subpopulations of adaptive immunity cells.

Additionally, animals who received FluVec-N or other vaccine formulations, including the empty vector, had a lower percentage of monocytes compared to the placebo control (Figure 5c). The low content of these cells may indicate their less intense migration from the peripheral blood in animals with a formed adaptive immune response. A similar explanation can be offered for NK cells, the level of which was also lower in vaccinated animals than in the control (Figure 5f).

Immunization with FluVec-N prevented an increase in eosinophils in the lungs during SARS-CoV-2 infection. In contrast, mice vaccinated with the inactivated SARS-CoV-2 showed a marked increase in the relative content of eosinophils in the lung tissue (Figure 5e). At the same time, in mice vaccinated using the prime–boost scheme, the relative content of eosinophils in the lung interstitium did not differ from that of other groups, including intact animals.

#### 3.3.2. Adaptive Immunity in Vaccinated Mice upon Subsequent Infection

**Figure 6:**
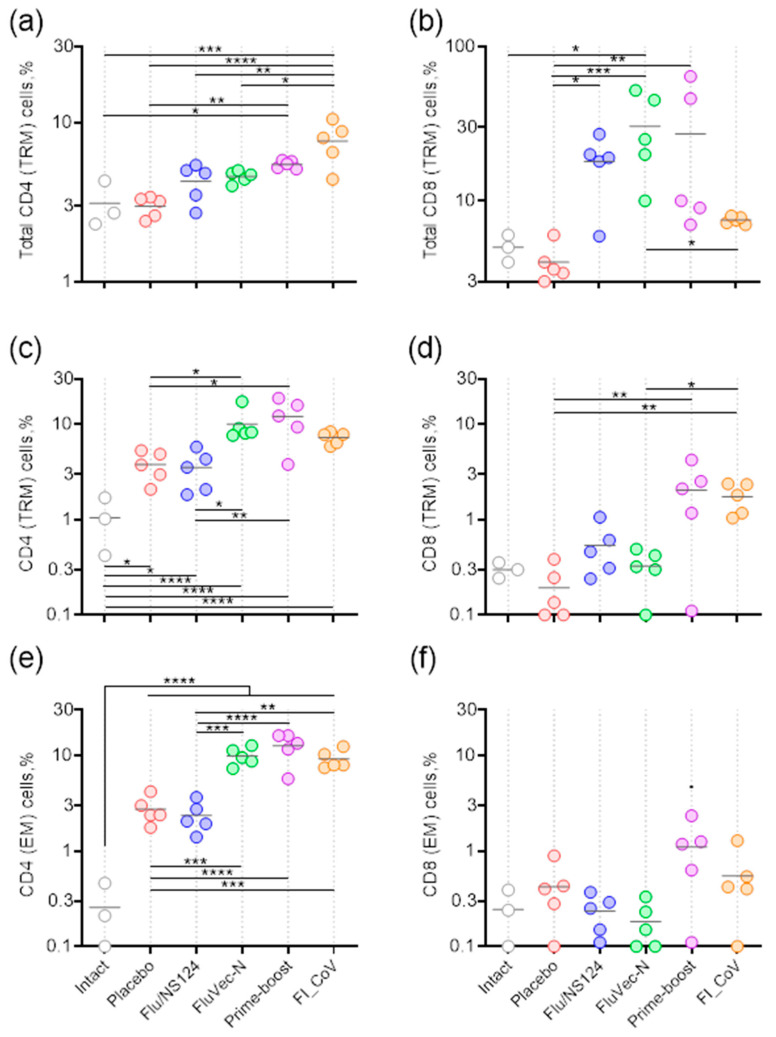
Main populations of CD4+ and CD8+ memory T lymphocytes in BALB/c mouse lung tissue 5 days after infection. (**a**,**b**) Total tissue-resident memory cells (CD4/CD8+CD44+CD62L-CD103+CD69+); (**c**,**d**) N-specific cytokine-producing tissue-resident memory cells; (**e**,**f**) N-specific cytokine-producing effector memory cells. Percentages of cells in the corresponding population are presented individually for each animal, and the horizontal line represents the group mean. An intact group is presented for comparison with normal non-infected mice. * *p* < 0.05, ** *p* < 0.01, *** *p* < 0.001, **** *p* < 0.0001 calculated using Tukey post-hoc test following one-way ANOVA applied to log values (*p* < 0.0001).

On day five after SARS-CoV-2 infection, the main populations of lung memory CD4+ and CD8+ T-lymphocytes were analyzed via flow cytometry (Figure 6). Mice that received the FluVec-N vector as a prime or boost vaccination showed a significant increase in the percentage of total CD8+ resident memory T lymphocytes (Trm) in the pulmonary interstitium. The proportion of Trm in these animals reached 60%. At the same time, no increase in Trm was observed in mice vaccinated with the inactivated coronavirus. The opposite effect was shown for total CD4+ Trm: mice vaccinated twice with the inactivated virus showed a more pronounced increase in the relative content of these cells compared to other groups (Figure 6a,b).

Virus-specific memory T cells were detected through inflammatory cytokine production (IFNγ, IL-2, TNFα) upon stimulation of isolated lung cells with the SARS-CoV-2 N-protein peptide pool. Mice immunized with FluVec-N twice or in the prime–boost regimen and mice vaccinated with formalin-inactivated coronavirus developed a pronounced virus-specific CD4+ immune response. The percentages of cytokine-producing effector memory (Tem) cells and CD4+ Trm were similar in these groups (Figure 6c,e). In addition, mice administered with FluVec-N in the prime–boost regimen tended to have higher percentages of antigen-specific CD8+ Tem and Trm lymphocytes (Figure 6d,f).

**Figure A5:**
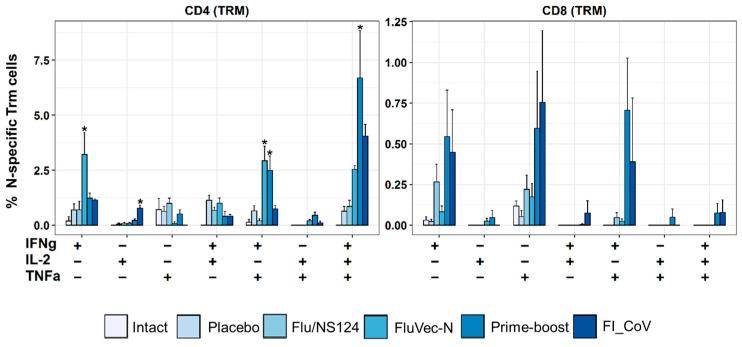
Relative content of individual subpopulations of cytokine-producing resident CD4+ and CD8+ T lymphocytes. Data obtained after subtracting background values of the relative content of cytokine-producing cells in the unstimulated control are presented. Statistical analysis was performed using ANOVA (*p* < 0.0001) followed by pairwise group comparison using Tukey’s test. * *p* < 0.05 marks significant differences with the placebo control group.

**Figure A6:**
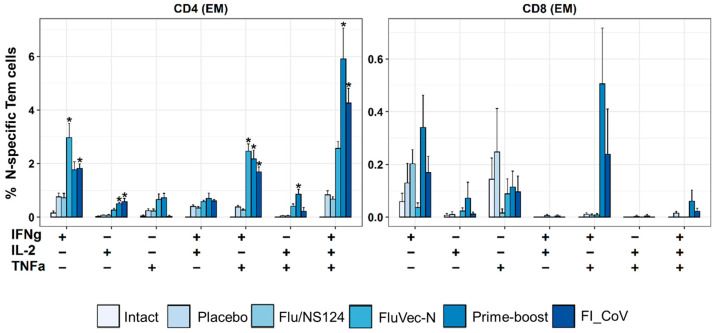
Relative content of individual subpopulations of cytokine-producing effector memory CD4+ and CD8+ T lymphocytes. Data obtained after subtracting background values of the relative content of cytokine-producing cells in the unstimulated control are presented. Statistical analysis was performed using ANOVA (*p* < 0.0001) followed by pairwise group comparison using Tukey’s test. * *p* < 0.05 marks significant differences with the placebo control group.

The subpopulation of polyfunctional CD4+ T lymphocytes predominated among antigen-specific Tem lymphocytes. No significant differences in individual subpopulations were found between the groups receiving the recombinant influenza vector with the insert (Figure A5). The subpopulation structure of antigen-specific resident T lymphocytes, and thus the nature of the immune response in experimental animals, corresponded to that of the general population of effector T lymphocytes (Figure A6).

## 4. Discussion

As a platform for the development of vector vaccines against respiratory pathogens, influenza vector offers several advantages. Replication-deficient influenza vector vaccines are considered safe, can stimulate protective antibodies and T-cell responses to the expressed foreign antigen, and can be produced using already approved technologies [12]. Several influenza vector vaccine candidates against COVID-19 have been described, with most vectors carrying a SARS-CoV-2 S-protein receptor-binding domain (RBD) insert [13,14,15,16,17]. Another influenza vector candidate explores the protective properties of a selected SARS-CoV-2 epitope cassette insert [18].

Although immunization with the RBD antigen could induce neutralizing antibodies able to prevent COVID-19 infection, this strategy has limitations due to the high mutation rate of the SARS-CoV-2 S-protein, which is the primary target of the antibody and T-cell response [19,20]. In our study, we constructed a recombinant influenza vector FluVec-N carrying a more conserved N-protein, which is the second main target of immune response to SARS-CoV-2 [19]. Our FluVec-N influenza vectors with the SARS-CoV-2 N-protein fragment insert have an attenuated phenotype as described earlier for an empty influenza vector with the truncated NS1 [21] and vectors with heterologous inserts from M. tuberculosis or respiratory syncytial virus (RSV) [22,23,24]. FluVec-N also provides a specific CD8+ immune response to the heterologous insert (SARS-CoV-2 N-protein).

As shown earlier, immunization of naïve animals with NS1-truncated influenza virus can provide regulatory signals restraining the inflammatory response upon subsequent influenza infection [25]. A similar mechanism underlies the protective properties of the dNS1-RBD influenza vector, a COVID-19 vaccine candidate, in hamsters. Intranasal vector delivery has been shown to induce trained immunity and tissue-resident memory T cells, restraining the inflammatory response during the SARS-CoV-2 challenge and reducing excessive immune-induced tissue damage [26]. An attractive idea was to explore whether the regulatory functions of the influenza virus with the truncated NS1 could be expanded to protect against heterologous pathogens when the influenza vector is used for vaccination in the context of suboptimal pre-existing immunity.

The important finding of our study is the protective effect of FluVec-N booster vaccination in the prevention of COVID-19 disease enhancement observed in primed Th2-prone mice. Mice primed with the aluminum-adjuvanted formalin-inactivated B.1 lineage SARS-CoV-2 and challenged with the antigenically distant beta variant virus developed eosinophilia in the lungs and increased inflammation—signs, similar to the well-known RSV vaccine-associated enhanced respiratory disease (VAERD) [27]. Similar immunopathology was previously observed in different models of SARS-CoV and MERS coronavirus infections [28,29] and was recently described in detail for BALB/c mouse and hamster models of SARS-CoV-2 [30,31]. Only partial reduction of COVID-19 vaccine-enhanced disease was shown by reboosting seropositive mice with an RIBI-adjuvanted pre-fusion stabilized recombinant spike protein vaccine [32]. FluVec-N immunization of mice primed with an inactivated vaccine completely prevented the disease enhancement, as indicated by the absence of weight loss and a significant reduction in eosinophilia compared to primed-only animals. Protection was associated with the formation of N-specific IgA in mouse lungs and the early activation of virus-specific effector and resident CD8+ lymphocytes in mouse lungs. The latter echoes the effect described earlier for influenza infection [25], confirming the immunoregulatory role of immunization with the NS1-truncated influenza vector for the subsequent immune response during the challenge.

Although no VAERD was observed in any clinical trial of COVID-19 vaccines, according to the WHO recommendations, the potential of enhanced disease should be considered when developing new vaccines against COVID-19 or introducing currently licensed vaccines in new target populations [33]. A recently published study showed that inactivated COVID-19 vaccines might increase the risk of Omicron infection, and at least one dose of a non-inactivated COVID-19 vaccine could be used to overcome the threat [34]. Our study demonstrated that the influenza vector-based COVID-19 vaccine could be the choice in the case of patients who experience suboptimal immune response to SARS-CoV-2 infection.

Another risk factor of enhanced disease is associated with a potential antibody-dependent enhancement (ADE) of infection. The ADE of SARS-CoV-2 infection was recently reported in serum samples from patients previously infected with MERS-CoV [35]. The risk of ADE was shown to be potentially linked to N-specific antibodies targeting the 152–172 amino acid fragment of the N-protein, which bears three amino acids (Q163, L167, and K169) conserved among SARS-CoV, MERS-CoV, and SARS-CoV-2 viruses. The proposed mechanism is an increase in inflammation and exacerbation of the cytokine storm by macrophages activated with N-protein-bound antibodies, as demonstrated by the increased secretion of IL-6 [36]. Previous studies of SARS-CoV vector vaccines showed that immunization with vaccinia virus vector or Venezuelan equine encephalitis virus replicon particles, bearing the virus N protein but not the Spike protein, was associated with immunopathology [37,38]. In the case of SARS-CoV-2 N-protein vaccines, there were no described signs of immunopathology, irrespective of the animal model or vaccine type [39,40]. Two COVID-19 vaccines based on the full-length recombinant N-protein reached clinical trials: Osivaxx has recently entered phase I (NCT06128382) [41], and Convacell showed 85% efficacy in Phase III clinical trials (NCT05726084) [42], demonstrating that the risk of N-linked ADE might be re-evaluated. In our study, the FluVec-N influenza vector was designed to express the C-terminal part of SARS-CoV-2 N-protein. The immunization with FluVec-N aims the immune response at targets outside of the 152–172 region of the N-protein and thus could reduce the risk of ADE. No signs of disease enhancement were observed in FluVec-N influenza vector immunized mice. Moreover, double FluVec-N immunization of naïve mice prevented pulmonary tissue damage and inflammation upon SARS-CoV-2 infection, thereby reflecting the absence of disease enhancement. A similar effect was observed in the empty vector group, which suggests a trained immunity response that may explain previous findings regarding some level of protection against SARS-CoV-2 with the empty Delta-NS1 influenza vector [43].

Regarding the occurrence and importance of the VAERD phenomenon, it would be an attractive goal to explore the potential of influenza vectors with truncated NS1 in preventing disease enhancement for other infections besides SARS-CoV-2. The next most obvious target could be the RSV infection, especially because the enhanced disease in the case of RSV is also associated with Th2-biases immunopathology [27,44]. We previously constructed influenza vectors with truncated NS1 coding for RSV F-protein fragments and demonstrated their safety and protective effect against RSV [24]. We were also able to demonstrate the potency of influenza virus NS vectors expressing different antigenic proteins of M. tuberculosis to induce strong Th1-type specific immune response and protection against virulent mycobacterial challenge in animal models [22,23,45]. The obtained results substantiated the clinical evaluation of the candidate influenza-vectored vaccines against RSV, SARS-CoV-2, and M. tuberculosis in healthy volunteers (NCT05970744, NCT05945498, NCT05696067).

Looking from the other side, there are human pathological conditions, accompanied by the type 2 immune response. One example is asthma, a common chronic airway disease, witch pathogenesis is linked to type 2 immune response [46]. Also, some groups of people, e.g., aged people or people living in areas with high prevalence of helminths invasions are at risk for dysregulation of the adaptive immune system and associated increase of the viral disease severity [47]. Prophylaxis of respiratory viral infections in these particular populations should take into account the impact of the immune status and in the case of Th2-bias, influenza-vectored vaccine might be the choice.

## 5. Conclusions

Our work describes the development of FluVec-N, an attenuated influenza vector with a truncated NS1 protein that carries a fragment of the SARS-CoV-2 N-protein, as a potential COVID-19 vaccine candidate. We have shown that double immunization with FluVec-N reduces SARS-CoV-2 disease severity in naïve mice. Moreover, boost immunization with FluVec-N protects seropositive Th2-prone mice against SARS-CoV-2 disease enhancement. Our study supports the potential of using prime and boost mucosal immunization with influenza vector vaccines to prevent respiratory diseases and associated immunopathology.

## 6. Patents

The results reported in the manuscript were used to fill the Russian Federation national patent application, Patent RU 2802058 priority date of 28 December 2022.

## Data Availability

The data presented in this study are available on request from the corresponding author. The data are not publicly available due to the institution’s policy.

## Supplementary Materials

The following supporting information can be downloaded at https://www.mdpi.com/article/10.3390/vaccines13010015/s1, The original images.

## Appendix A

After eliminating FSC-A/FSC-H doublets and isolating a population of living single cells based on light scattering characteristics (FSC-A/SSC-A-Cells) and Zombie Red (ZR) dye binding, immunocytes were gated based on the presence of the CD45 marker (CD45+). Upon further analysis, the following cell populations were isolated: neutrophils—SSChiCD45+Ly6G+; monocytes—MHCII-CD64+CD11b+; alveolar macrophages—MHCII+CD64+CD11c+CD11b−; interstitial macrophages—MHCII+CD64+CD11b+CD11c+/−; dendritic cells—MHCII+CD64−CD24+ (CD11b+CD103+, CD11b−CD103+, CD11b+CD103−, CD11b−CD103−).

Based on forward and side scattering (FSC/SSC) characteristics and Zombie Red fluorescence, nonviable cells were excluded from the analysis. The living cell population was divided into two main subpopulations of T lymphocytes: T helper cells (CD4+) and cytotoxic T cells (CD8+). Based on the presence of the CD44 and CD62L markers, subpopulations of central (CM) and effector (EM) memory T cells were identified. The effector memory T cell population was further characterized by the production of intracellular cytokines IFNγ, TNFα, and IL2.

## References

[1] Holder J. Tracking Coronavirus Vaccinations Around the World. Updated March 13, 2023. Electronic Resource. https://www.nytimes.com/interactive/2021/world/covid-vaccinations-tracker.html.

[2] Bergeri I., Whelan M.G., Ware H., Subissi L., Nardone A., Lewis H.C., Li Z., Ma X., Valenciano M., Cheng B. (2022). Global SARS-CoV-2 seroprevalence from January 2020 to April 2022: A systematic review and meta-analysis of standardized population-based studies. PLoS Med..

[3] Bobrovitz N., Arora R.K., Cao C., Boucher E., Liu M., Donnici C., Yanes-Lane M., Whelan M., Perlman-Arrow S., Chen J. (2021). Global seroprevalence of SARS-CoV-2 antibodies: A systematic review and meta-analysis. PLoS ONE.

[4] Rostami A., Sepidarkish M., Fazlzadeh A., Mokdad A.H., Sattarnezhad A., Esfandyari S., Riahi S.M., Mollalo A., Dooki M.E., Bayani M. (2021). Update on SARS-CoV-2 seroprevalence: Regional and worldwide. Clin. Microbiol. Infect..

[5] Global COVID-19 Vaccination Strategy in a Changing World: July 2022 Update. https://www.who.int/publications/m/item/global-covid-19-vaccination-strategy-in-a-changing-world--july-2022-update.

[6] Vasilyev K., Shurygina A.P., Zabolotnykh N., Sergeeva M., Romanovskaya-Romanko E., Pulkina A., Buzitskaya J., Dogonadze M.Z., Vinogradova T.I., Stukova M.A. (2021). Enhancement of the Local CD8+ T-Cellular Immune Response to *Mycobacterium tuberculosis* in BCG-Primed Mice after Intranasal Administration of Influenza Vector Vaccine Carrying TB10.4 and HspX Antigens. Vaccines.

[7] Hoffmann E., Neumann G., Kawaoka Y., Hobom G., Webster R.G. (2000). A DNA transfection system for generation of influenza a virus from eight plasmids. Proc. Natl. Acad. Sci. USA.

[8] Krivitskaya V.Z., Sorokin E.V., Tsareva T.R., Sergeeva M.V., Kadyrova R.A., Romanovskaya-Roman’ko E.A., Shaldzhyan A.A., Petrov S.V., Petrova E.R., Konovalova N.I. (2018). Generation and Characterization of the Monoclonal Antibody Panel Specific to the NS1 Protein of the Influenza A Virus. Appl. Biochem. Microbiol..

[9] Zhang T., Magazine N., McGee M.C., Carossino M., Veggiani G., Kousoulas K.G., August A., Huang W. (2024). Th2 and Th17-associated immunopathology following SARS-CoV-2 breakthrough infection in Spike-vaccinated ACE2-humanized mice. J. Med. Virol..

[10] Kant R., Kareinen L., Smura T., Freitag T.L., Jha S.K., Alitalo K., Meri S., Sironen T., Saksela K., Strandin T. (2021). Common Laboratory Mice Are Susceptible to Infection with the SARS-CoV-2 Beta Variant. Viruses.

[11] Montagutelli X., Prot M., Levillayer L., Salazar E.B., Jouvion G., Conquet L., Donati F., Albert M., Gambaro F., Behillil S. (2021). The B1.351 and P.1 variants extend SARS-CoV-2 host range to mice. BioRxiv.

[12] Gerlach T., Elbahesh H., Saletti G., Rimmelzwaan G.F. (2019). Recombinant influenza A viruses as vaccine vectors. Expert Rev. Vaccines.

[13] Zhou R., Wang P., Wong Y.C., Xu H., Lau S.Y., Liu L., Mok B.W.Y., Peng Q., Liu N., Woo K.F. (2022). Nasal prevention of SARS-CoV-2 infection by intranasal influenza-based boost vaccination in mouse models. EBioMedicine.

[14] Zhao Y., Zhao L., Li Y., Liu Q., Deng L., Lu Y., Zhang X., Li S., Ge J., Bu Z. (2022). An influenza virus vector candidate vaccine stably expressing SARS-CoV-2 receptor-binding domain produces high and long-lasting neutralizing antibodies in mice. Vet. Microbiol..

[15] Deng S., Liu Y., Tam R.C.Y., Chen P., Zhang A.J., Mok B.W.Y., Long T., Kukic A., Zhou R., Xu H. (2023). An intranasal influenza virus-vectored vaccine prevents SARS-CoV-2 replication in respiratory tissues of mice and hamsters. Nat. Commun..

[16] Loes A.N., Gentles L.E., Greaney A.J., Crawford K.H.D., Bloom J.D. (2020). Attenuated Influenza Virions Expressing the SARS-CoV-2 Receptor-Binding Domain Induce Neutralizing Antibodies in Mice. Viruses.

[17] Stepanova E., Isakova-Sivak I., Mezhenskaya D., Niskanen S., Matyushenko V., Bazhenova E., Rak A., Wong P.F., Prokopenko P., Kotomina T. (2024). Expression of the SARS-CoV-2 receptor-binding domain by live attenuated influenza vaccine virus as a strategy for designing a bivalent vaccine against COVID-19 and influenza. Virol. J..

[18] Isakova-Sivak I., Stepanova E., Matyushenko V., Niskanen S., Mezhenskaya D., Bazhenova E., Krutikova E., Kotomina T., Prokopenko P., Neterebskii B. (2022). Development of a T Cell-Based COVID-19 Vaccine Using a Live Attenuated Influenza Vaccine Viral Vector. Vaccines.

[19] Shrock E., Fujimura E., Kula T., Timms R.T., Lee I.H., Leng Y., Robinson M.L., Sie B.M., Li M.Z., Chen Y. (2020). Viral epitope profiling of COVID-19 patients reveals cross-reactivity and correlates of severity. Science.

[20] Peng Y., Mentzer A.J., Liu G., Yao X., Yin Z., Dong D., Dejnirattisai W., Rostron T., Supasa P., Liu C. (2020). Broad and strong memory CD4^+^ and CD8^+^ T cells induced by SARS-CoV-2 in UK convalescent individuals following COVID-19. Nat. Immunol..

[21] Egorov A., Brandt S., Sereinig S., Romanova J., Ferko B., Katinger D., Grassauer A., Alexandrova G., Katinger H., Muster T. (1998). Transfectant influenza A viruses with long deletions in the NS1 protein grow efficiently in Vero cells. J. Virol..

[22] Sereinig S., Stukova M., Zabolotnyh N., Ferko B., Kittel C., Romanova J., Vinogradova T., Katinger H., Kiselev O., Egorov A. (2006). Influenza virus NS vectors expressing the Mycobacterium tuberculosis ESAT-6 protein induce CD4+ Th1 immune response and protect animals against tuberculosis challenge. Clin. Vaccine Immunol..

[23] Sergeeva M., Romanovskaya-Romanko E., Zabolotnyh N., Pulkina A., Vasilyev K., Shurigina A.P., Buzitskaya J., Zabrodskaya Y., Fadeev A., Vasin A. (2021). Mucosal Influenza Vector Vaccine Carrying TB10.4 and HspX Antigens Provides Protection against *Mycobacterium tuberculosis* in Mice and Guinea Pigs. Vaccines.

[24] Pulkina A., Vasilyev K., Muzhikyan A., Sergeeva M., Romanovskaya-Romanko E., Shurygina A.P., Shuklina M., Vasin A., Stukova M., Egorov A. (2023). IgGκ Signal Peptide Enhances the Efficacy of an Influenza Vector Vaccine against Respiratory Syncytial Virus Infection in Mice. Int. J. Mol. Sci..

[25] Vasilyev K., Shurygina A.P., Sergeeva M., Stukova M., Egorov A. (2021). Intranasal Immunization with the Influenza A Virus Encoding Truncated NS1 Protein Protects Mice from Heterologous Challenge by Restraining the Inflammatory Response in the Lungs. Microorganisms.

[26] Zhang L., Jiang Y., He J., Chen J., Qi R., Yuan L., Shao T., Zhao H., Chen C., Chen Y. (2023). Intranasal influenza-vectored COVID-19 vaccine restrains the SARS-CoV-2 inflammatory response in hamsters. Nat. Commun..

[27] Openshaw P.J., Tregoning J.S. (2005). Immune responses and disease enhancement during respiratory syncytial virus infection. Clin. Microbiol. Rev..

[28] Tseng C.T., Sbrana E., Iwata-Yoshikawa N., Newman P.C., Garron T., Atmar R.L., Peters C.J., Couch R.B. (2012). Immunization with SARS coronavirus vaccines leads to pulmonary immunopathology on challenge with the SARS virus. PLoS ONE.

[29] Agrawal A.S., Tao X., Algaissi A., Garron T., Narayanan K., Peng B.H., Couch R.B., Tseng C.T. (2016). Immunization with inactivated Middle East Respiratory Syndrome coronavirus vaccine leads to lung immunopathology on challenge with live virus. Hum. Vaccines Immunother..

[30] Ebenig A., Muraleedharan S., Kazmierski J., Todt D., Auste A., Anzaghe M., Gömer A., Postmus D., Gogesch P., Niles M. (2022). Vaccine-associated enhanced respiratory pathology in COVID-19 hamsters after T_H_2-biased immunization. Cell Rep..

[31] Iwata-Yoshikawa N., Shiwa N., Sekizuka T., Sano K., Ainai A., Hemmi T., Kataoka M., Kuroda M., Hasegawa H., Suzuki T. (2022). A lethal mouse model for evaluating vaccine-associated enhanced respiratory disease during SARS-CoV-2 infection. Sci. Adv..

[32] Dillard J.A., Taft-Benz S.A., Knight A.C., Anderson E.J., Pressey K.D., Parotti B., Martinez S.A., Diaz J.L., Sarkar S., Madden E.A. (2024). Adjuvant-dependent impact of inactivated SARS-CoV-2 vaccines during heterologous infection by a SARS-related coronavirus. Nat. Commun..

[33] WHO Target Product Profiles for COVID-19 Vaccines, April 2022. https://www.who.int/publications/m/item/who-target-product-profiles-for-covid-19-vaccines.

[34] Xiong H., Meng X., Song Y., Zhong J., Liu S., Zhu X., Ye X., Zhong Y., Zhang D. (2024). Have Previous COVID-19 Vaccinations Shaped the Potential Enhancing Infection of Variant Strains?. Vaccines.

[35] Thomas S., Smatti M.K., Alsulaiti H., Zedan H.T., Eid A.H., Hssain A.A., Abu Raddad L.J., Gentilcore G., Ouhtit A., Althani A.A. (2024). Antibody-dependent enhancement (ADE) of SARS-CoV-2 in patients exposed to MERS-CoV and SARS-CoV-2 antigens. J. Med. Virol..

[36] Nakayama E.E., Shioda T. (2023). SARS-CoV-2 Related Antibody-Dependent Enhancement Phenomena In Vitro and In Vivo. Microorganisms.

[37] Deming D., Sheahan T., Heise M., Yount B., Davis N., Sims A., Suthar M., Harkema J., Whitmore A., Pickles R. (2006). Vaccine efficacy in senescent mice challenged with recombinant SARS-CoV bearing epidemic and zoonotic spike variants. PLoS Med..

[38] Yasui F., Kai C., Kitabatake M., Inoue S., Yoneda M., Yokochi S., Kase R., Sekiguchi S., Morita K., Hishima T. (2008). Prior immunization with severe acute respiratory syndrome (SARS)-associated coronavirus (SARS-CoV) nucleocapsid protein causes severe pneumonia in mice infected with SARS-CoV. J. Immunol..

[39] Rak A., Isakova-Sivak I., Rudenko L. (2023). Overview of Nucleocapsid-Targeting Vaccines against COVID-19. Vaccines.

[40] Egorov A., Krokhin A.A., Leneva I.A., Korabliov P.V., Loiteris P., Nebolsin V.E. (2024). Single intranasal immunization with a high dose of influenza vector protects against infection with heterologous influenza virus and SARS-CoV-2 in ferrets and hamsters. Microbiol. Indep. Res. J..

[41] Primard C., Monchâtre-Leroy E., Del Campo J., Valsesia S., Nikly E., Chevandier M., Boué F., Servat A., Wasniewski M., Picard-Meyer E. (2023). OVX033, a nucleocapsid-based vaccine candidate, provides broad-spectrum protection against SARS-CoV-2 variants in a hamster challenge model. Front. Immunol..

[42] Rabdano S.O., Ruzanova E.A., Vertyachikh A.E., Teplykh V.A., Emelyanova A.B., Rudakov G.O., Arakelov S.A., Pletyukhina I.V., Saveliev N.S., Lukovenko A.A. (2024). N-protein vaccine is effective against COVID-19: Phase 3, randomized, double-blind, placebo-controlled clinical trial. J. Infect..

[43] Rathnasinghe R., Salvatore M., Zheng H., Jangra S., Kehrer T., Mena I., Schotsaert M., Muster T., Palese P., García-Sastre A. (2021). Interferon mediated prophylactic protection against respiratory viruses conferred by a prototype live attenuated influenza virus vaccine lacking non-structural protein 1. Sci. Rep..

[44] Eichinger K.M., Kosanovich J.L., Perkins T.N., Oury T.D., Petrovsky N., Marshall C.P., Yondola M.A., Empey K.M. (2022). Prior respiratory syncytial virus infection reduces vaccine-mediated Th2-skewed immunity, but retains enhanced RSV F-specific CD8 T cell responses elicited by a Th1-skewing vaccine formulation. Front. Immunol..

[45] Shurygina A.P., Zabolotnykh N., Vinogradova T., Khairullin B., Kassenov M., Nurpeisova A., Sarsenbayeva G., Sansyzbay A., Vasilyev K., Buzitskaya J. (2023). Preclinical Evaluation of TB/FLU-04L-An Intranasal Influenza Vector-Based Boost Vaccine against Tuberculosis. Int. J. Mol. Sci..

[46] Harker J.A., Lloyd C.M. (2023). T helper 2 cells in asthma. J. Exp. Med..

[47] Howard F.H.N., Kwan A., Winder N., Mughal A., Collado-Rojas C., Muthana M. (2022). Understanding Immune Responses to Viruses-Do Underlying Th1/Th2 Cell Biases Predict Outcome?. Viruses.

