# Supplementary figures and images for: Mucosal Immunization with an Influenza Vector Carrying SARS-CoV-2 N Protein Protects Naïve Mice and Prevents Disease Enhancement in Seropositive Th2-Prone Mice
Source: Vaccines (Basel). 2024 Dec 28;13(1):15. doi: 10.3390/vaccines13010015 (PMC11769390; doi:10.3390/vaccines13010015)

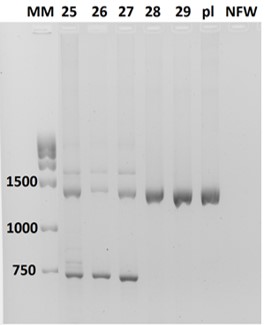

Supplement: Supplementary file 1 [file vaccines-13-00015-s001.zip › Figure A3.1.jpg]

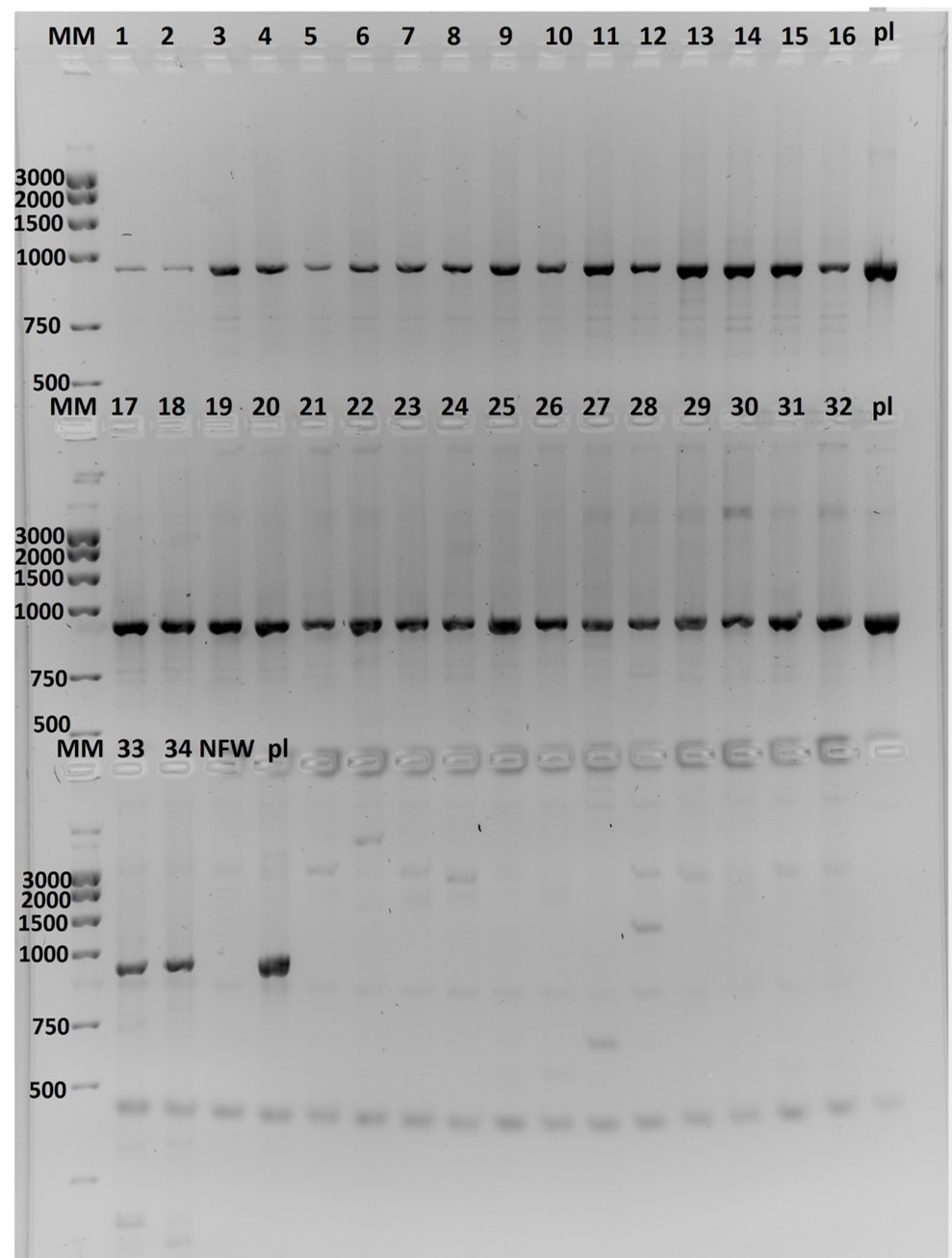

Supplement: Supplementary file 1 [file vaccines-13-00015-s001.zip › Figure A3.2.jpg]

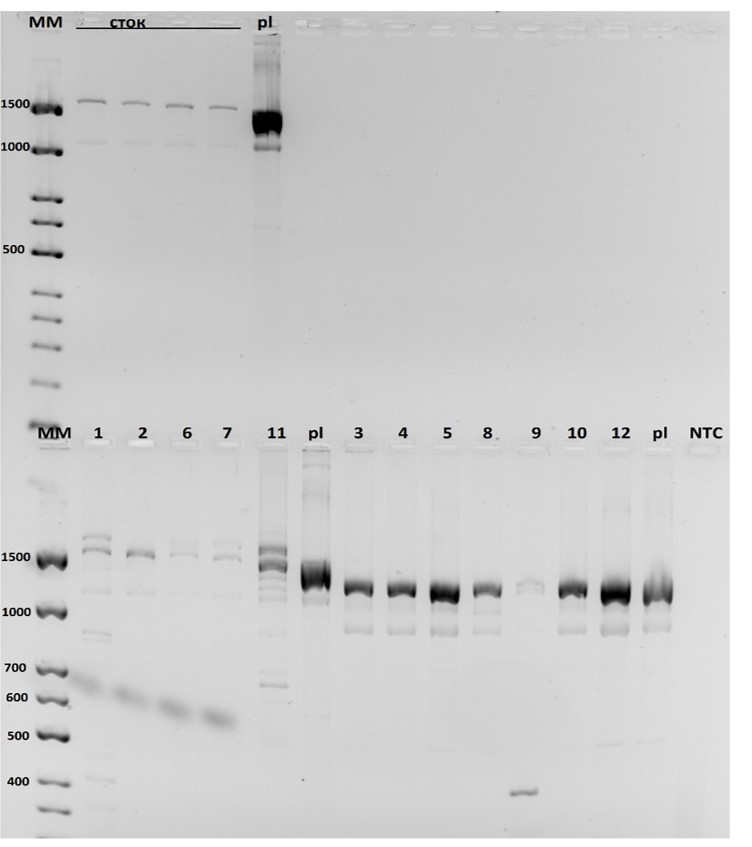

Supplement: Supplementary file 1 [file vaccines-13-00015-s001.zip › Figure A3.3.jpg]

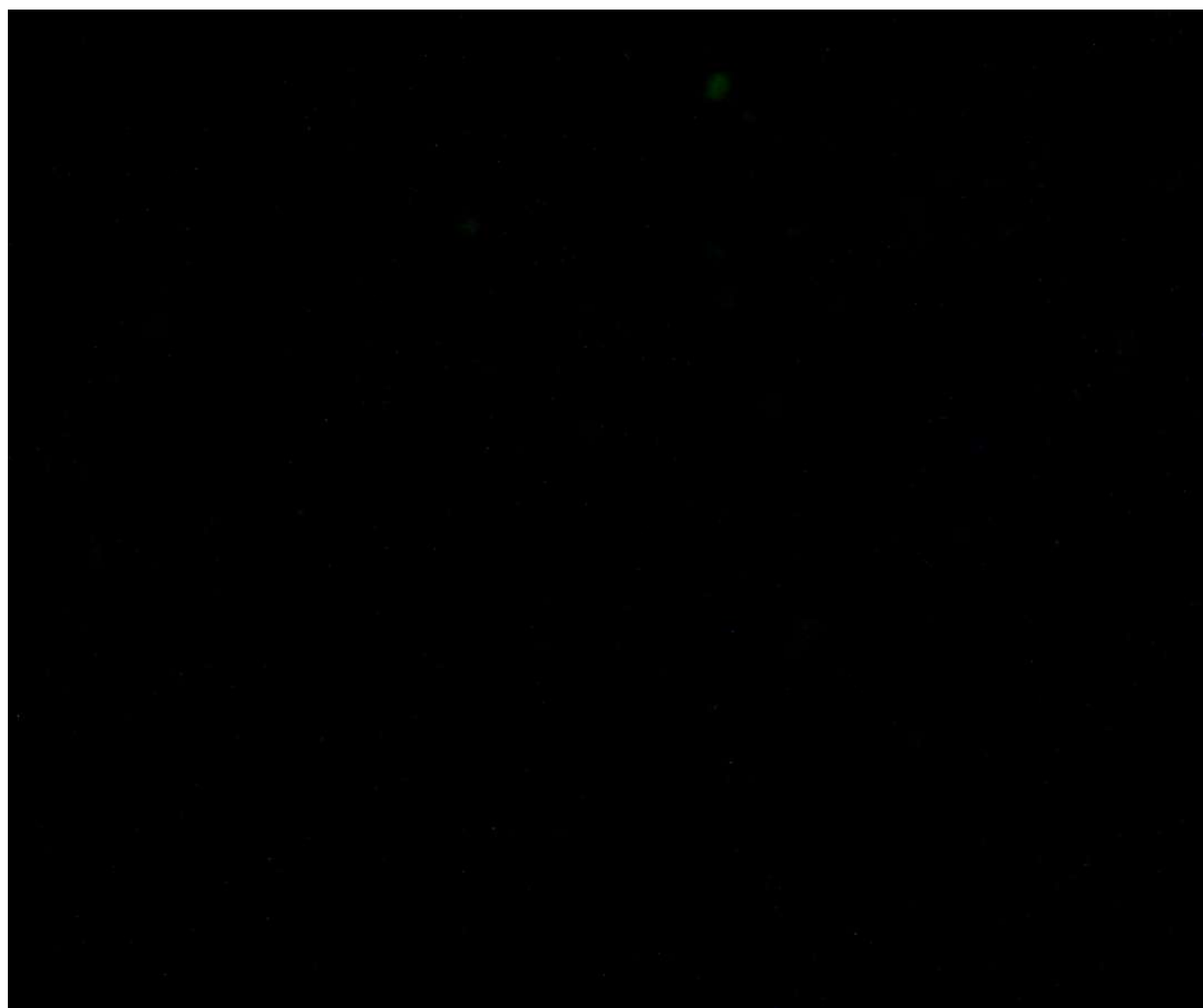

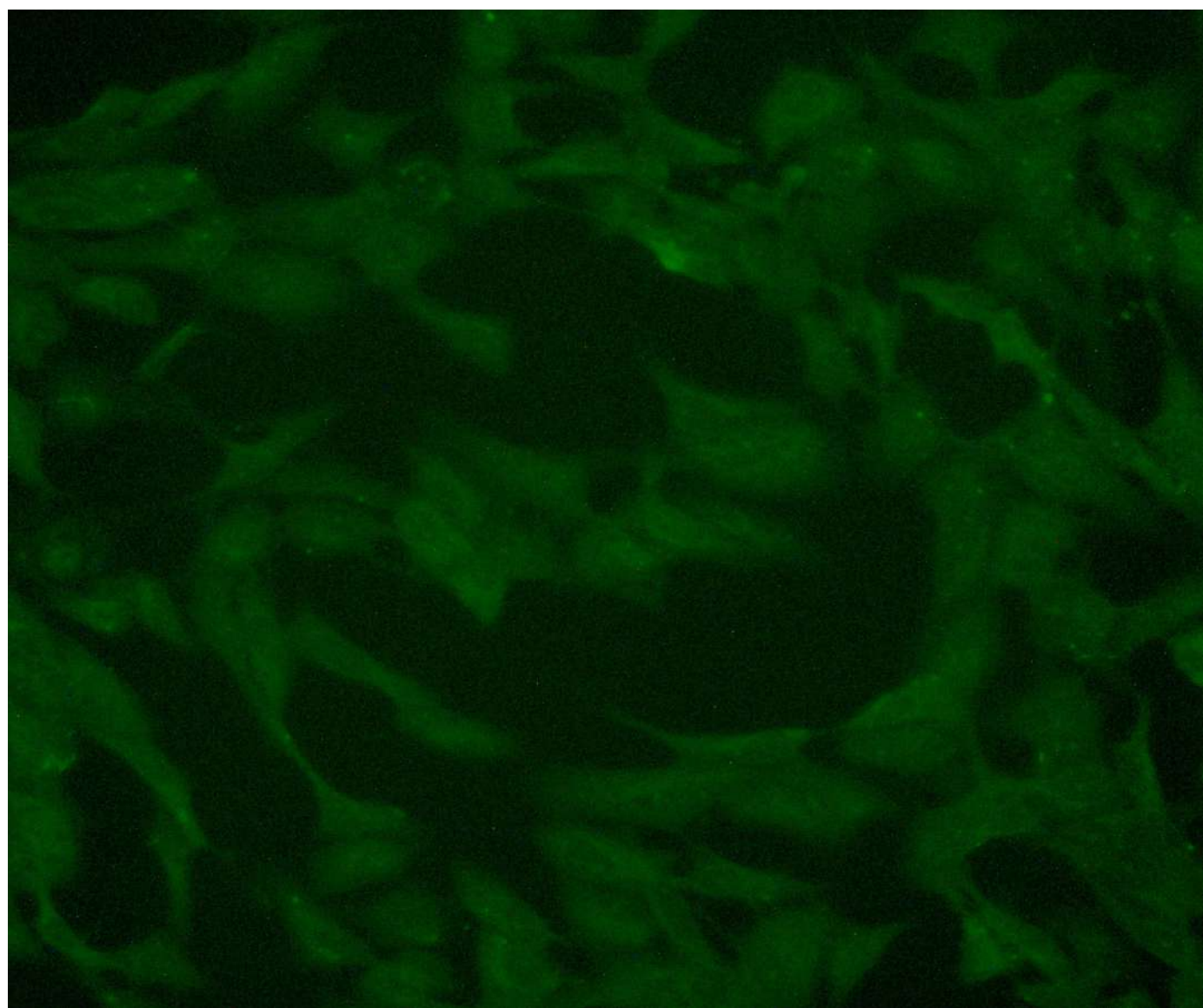

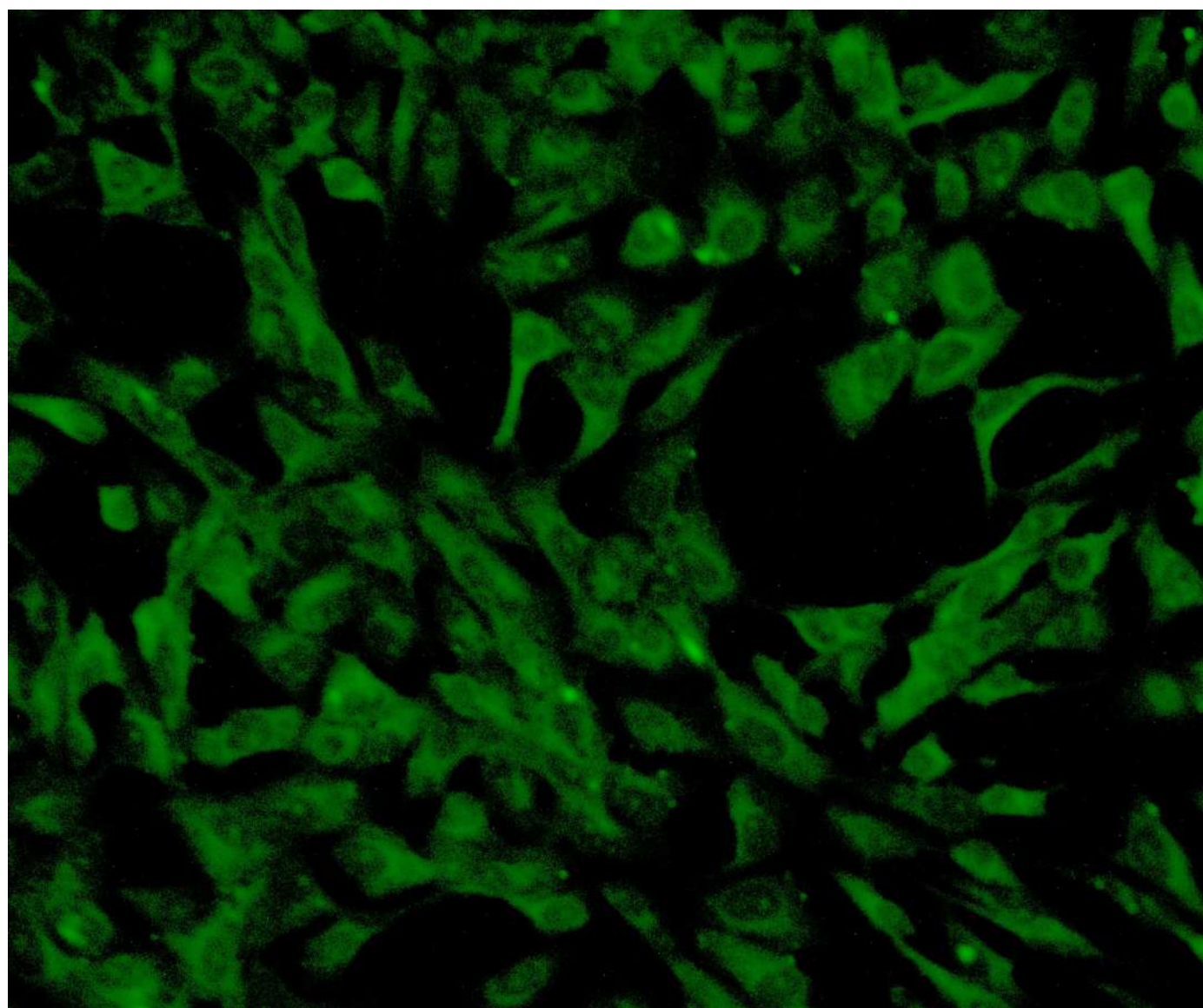

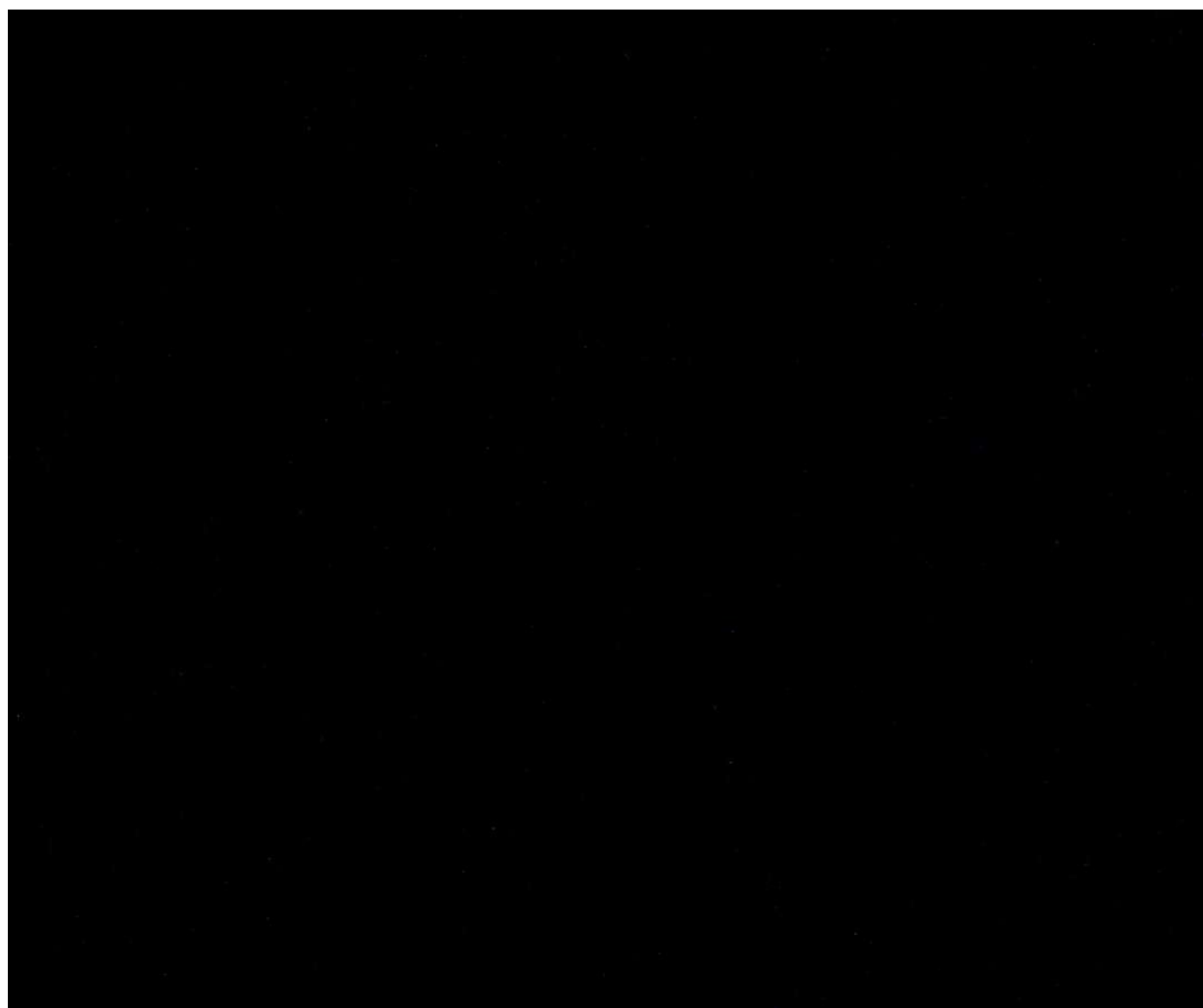

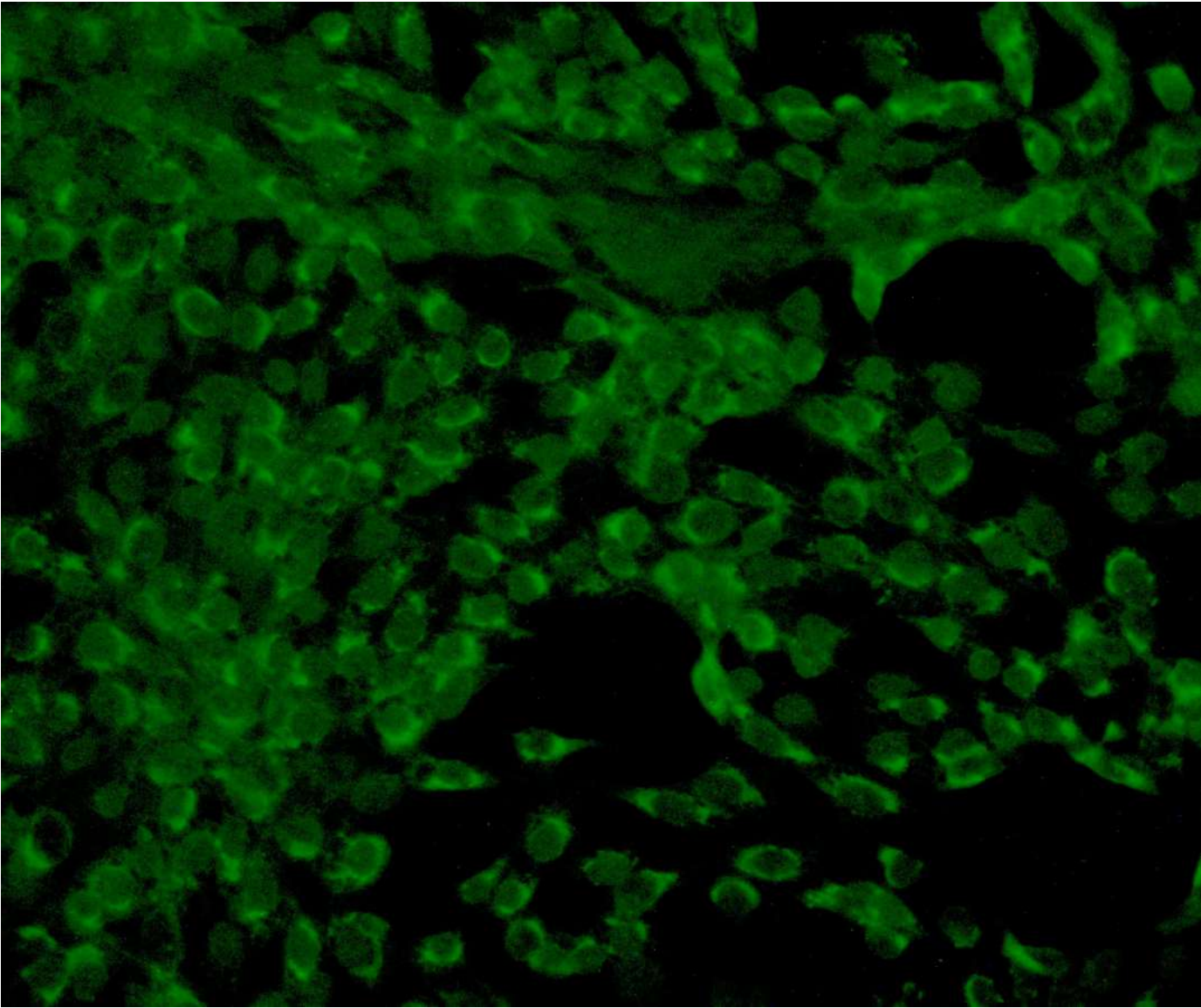

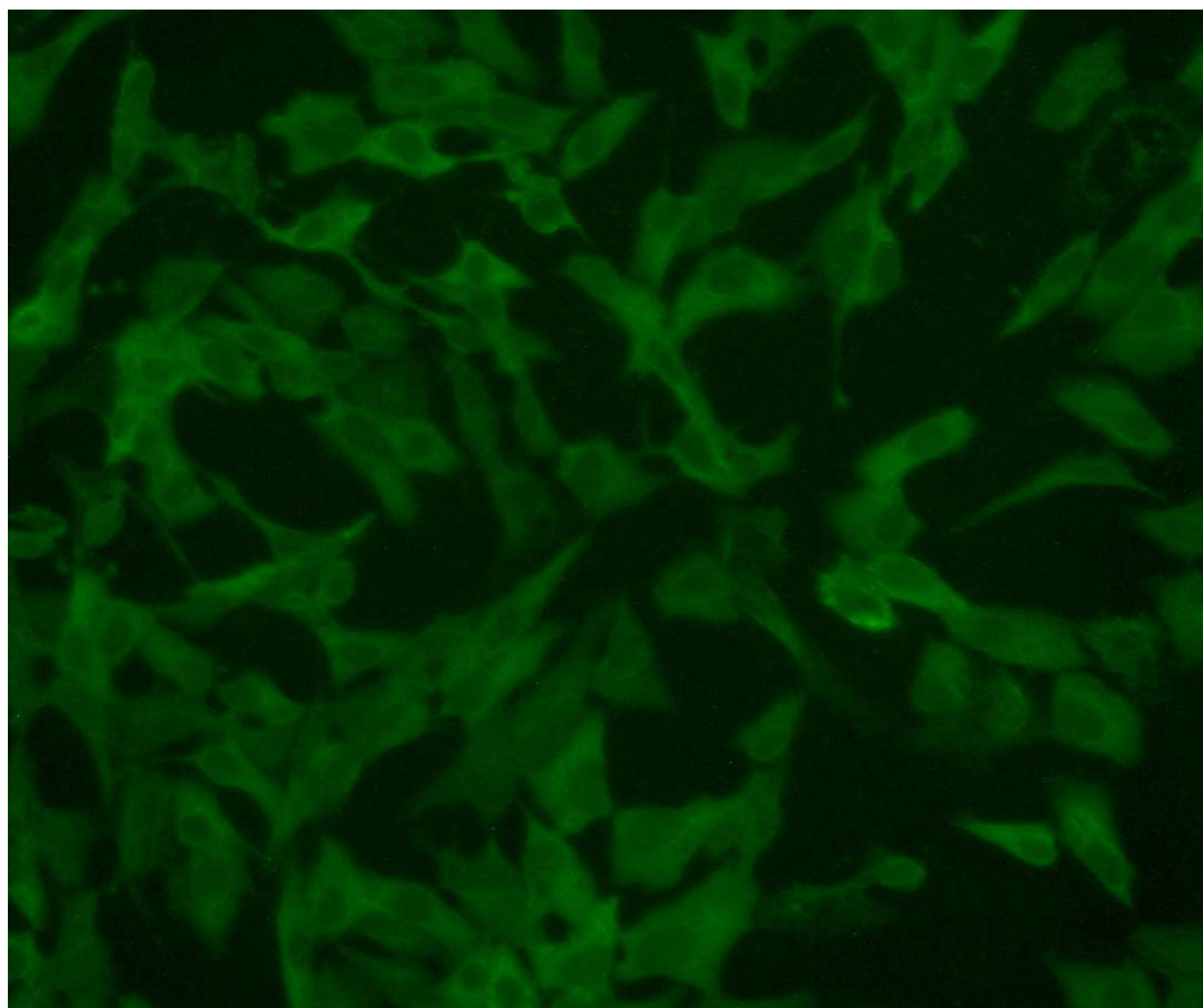

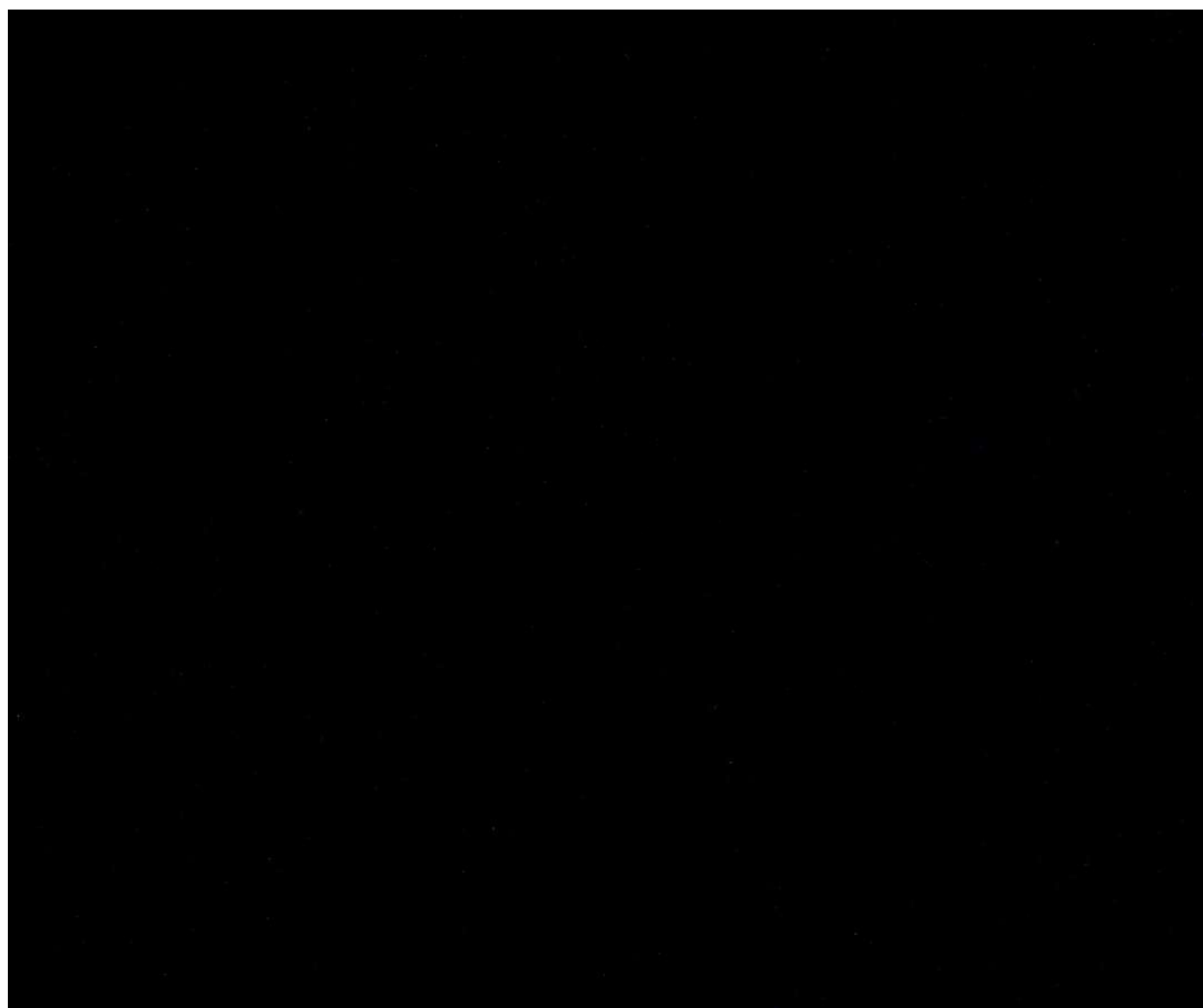

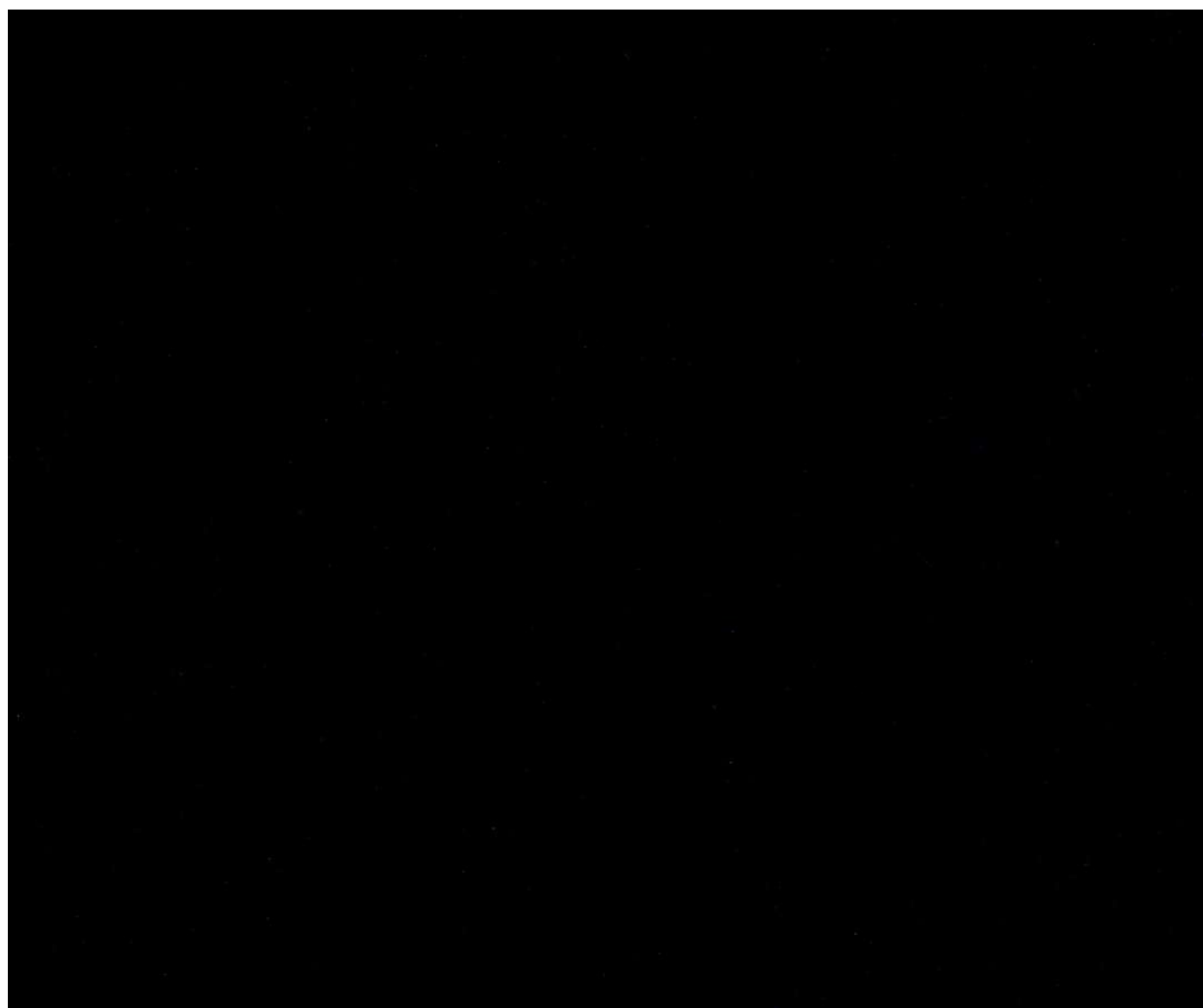

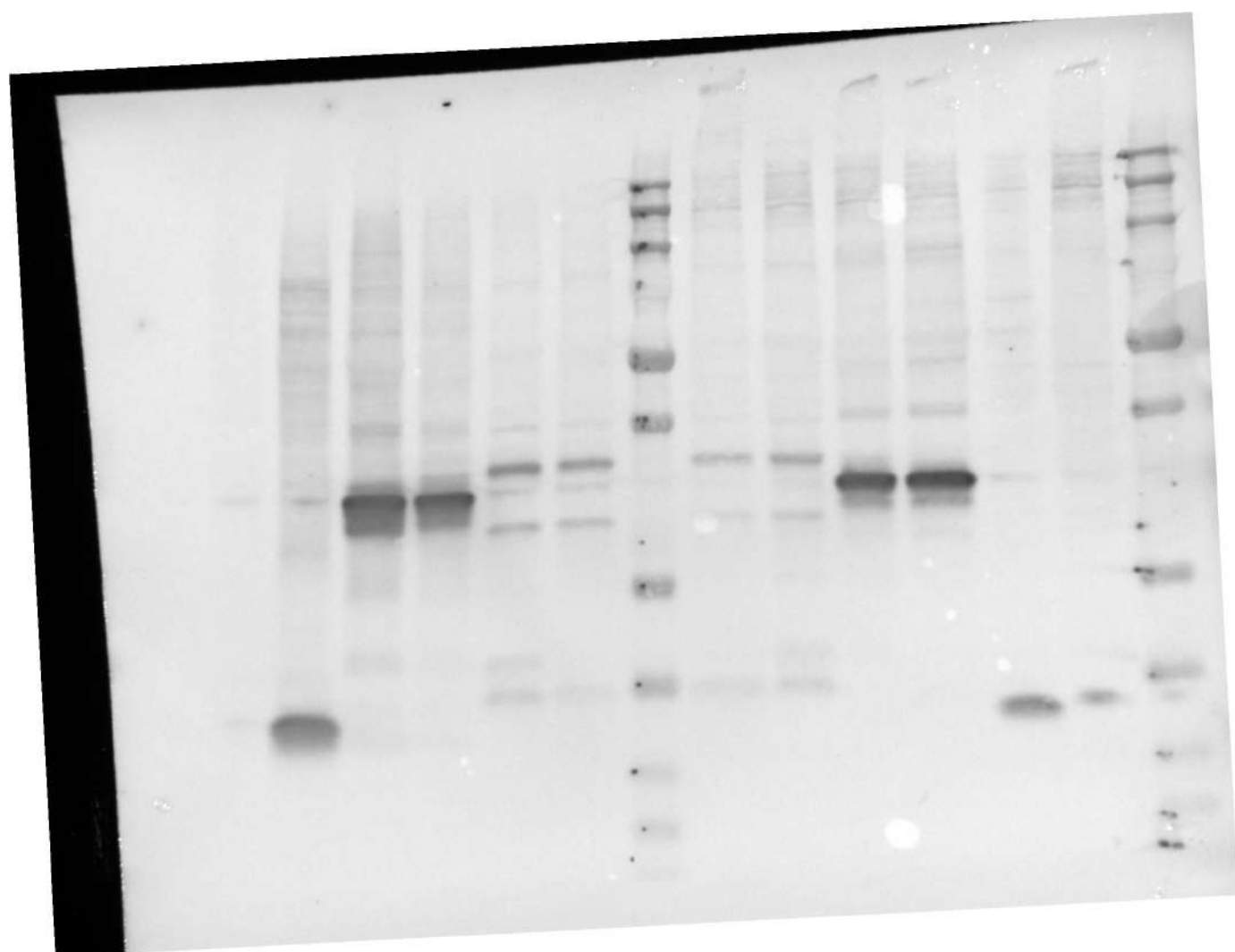

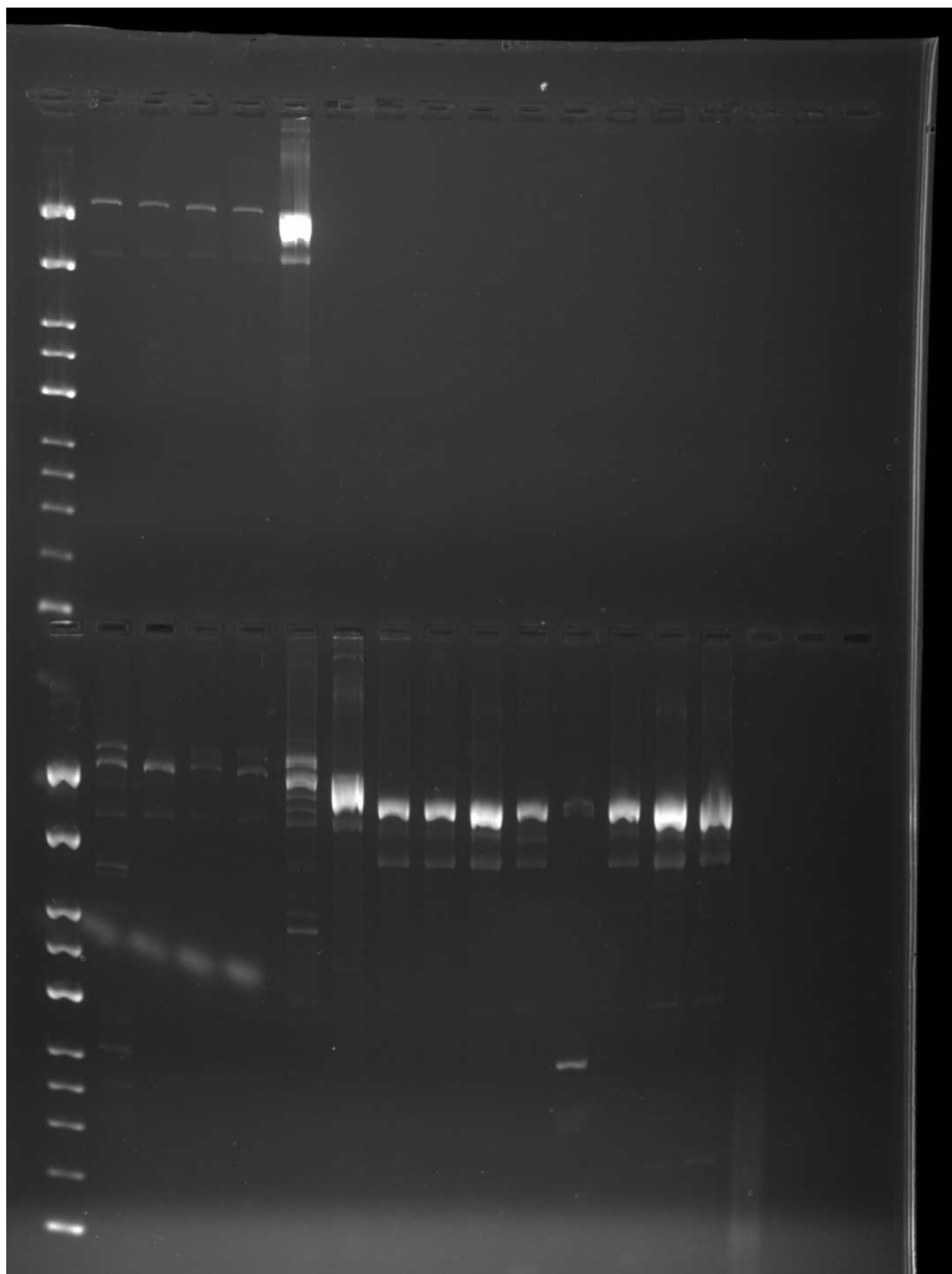

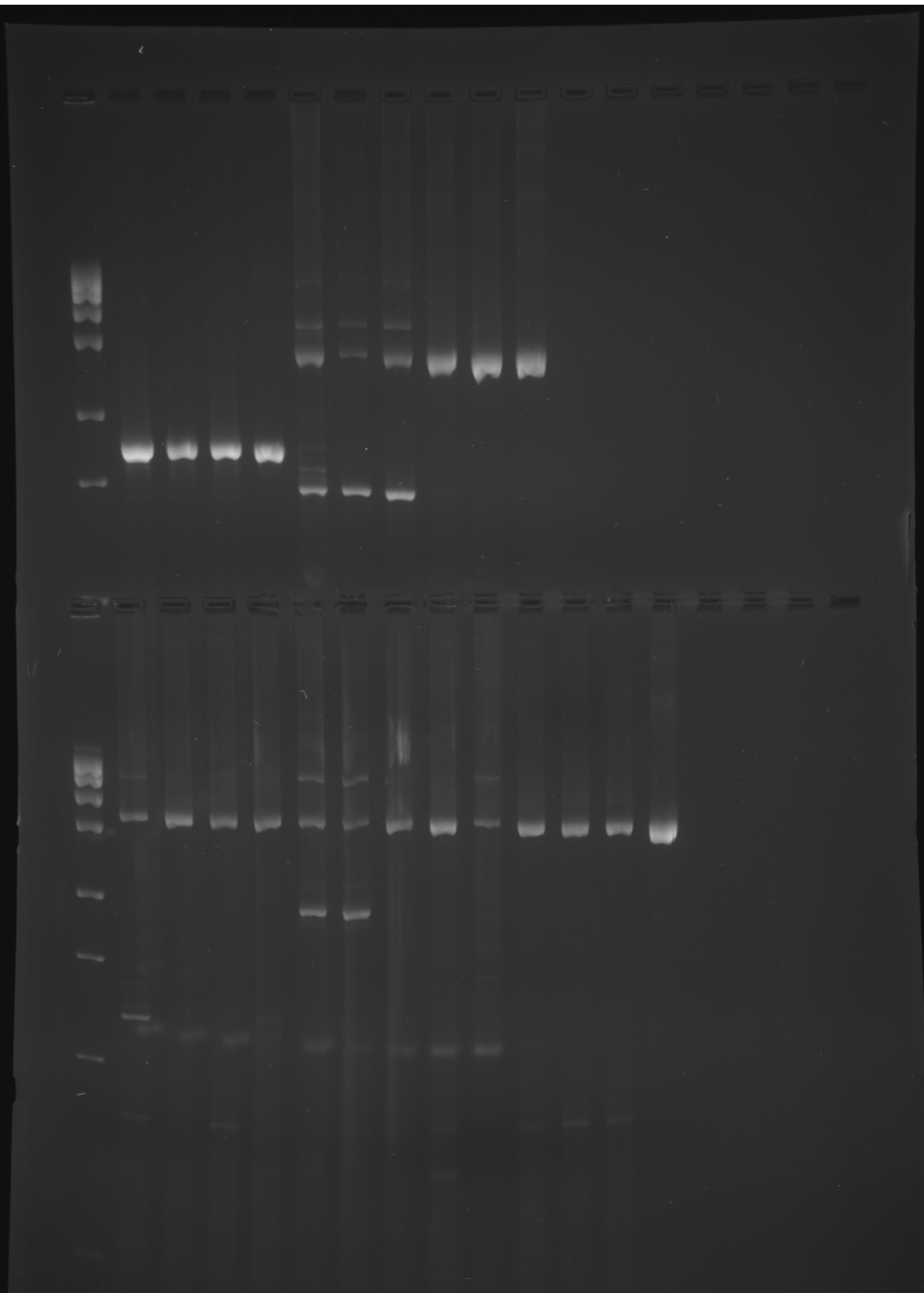

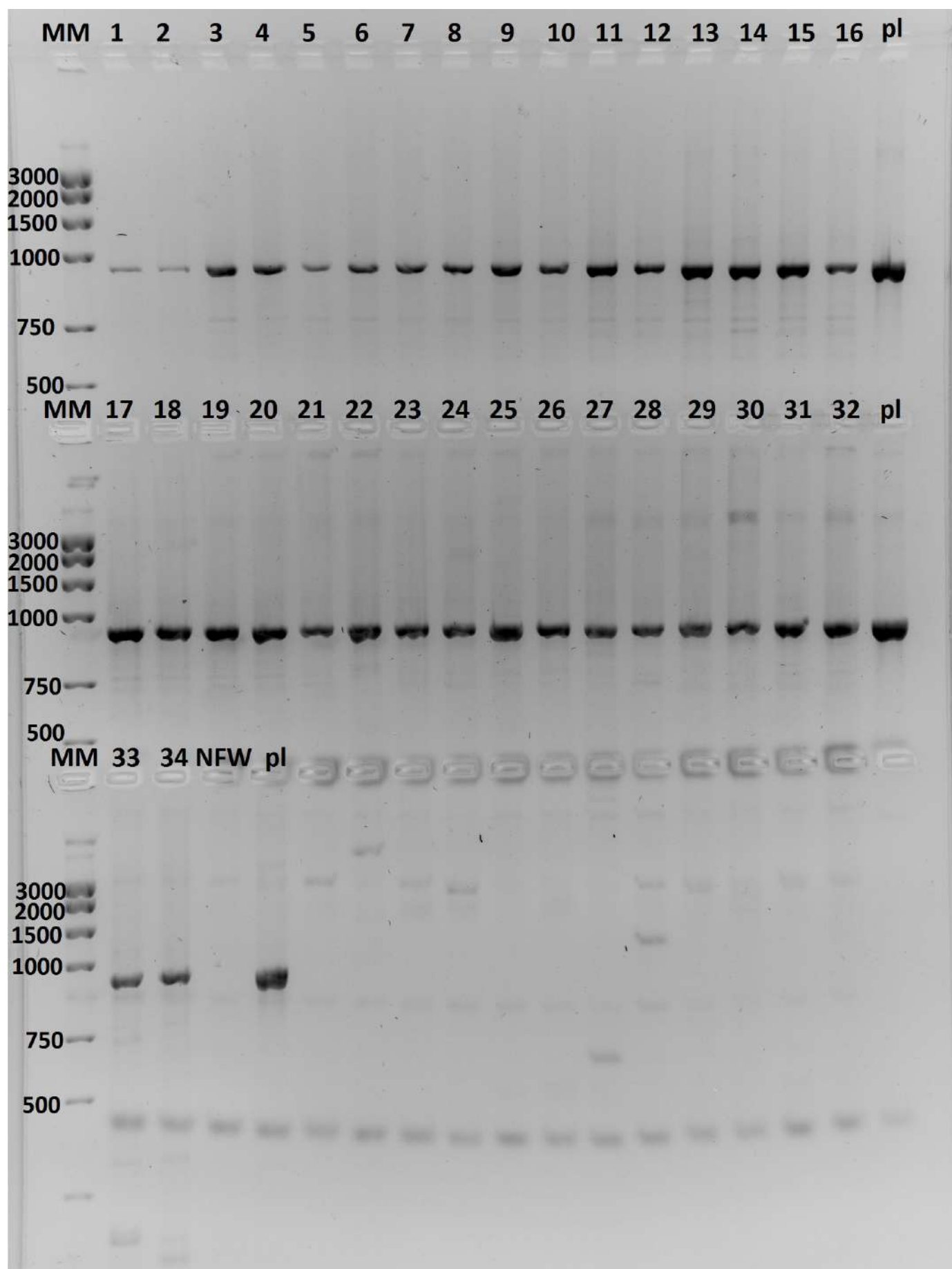

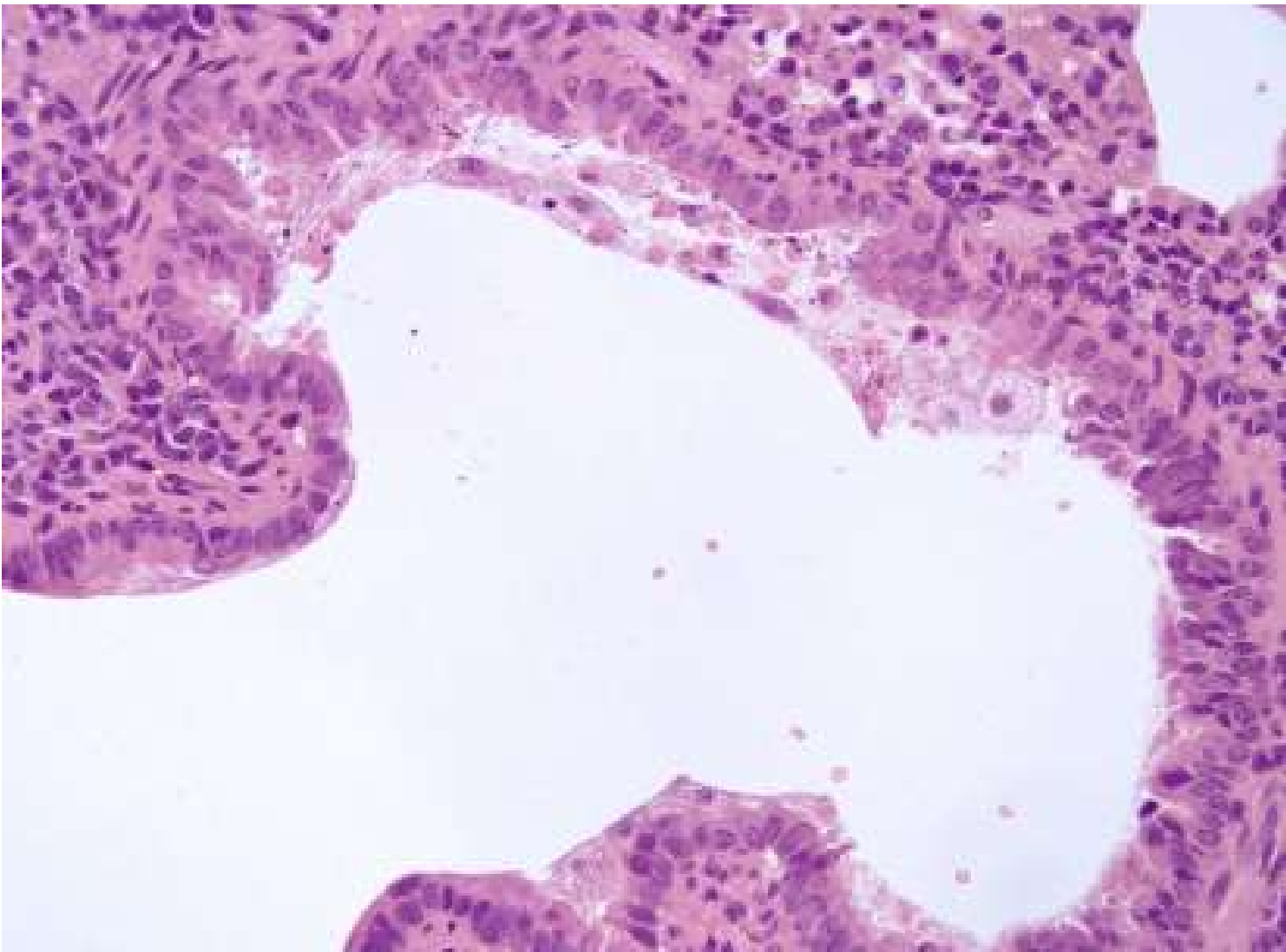

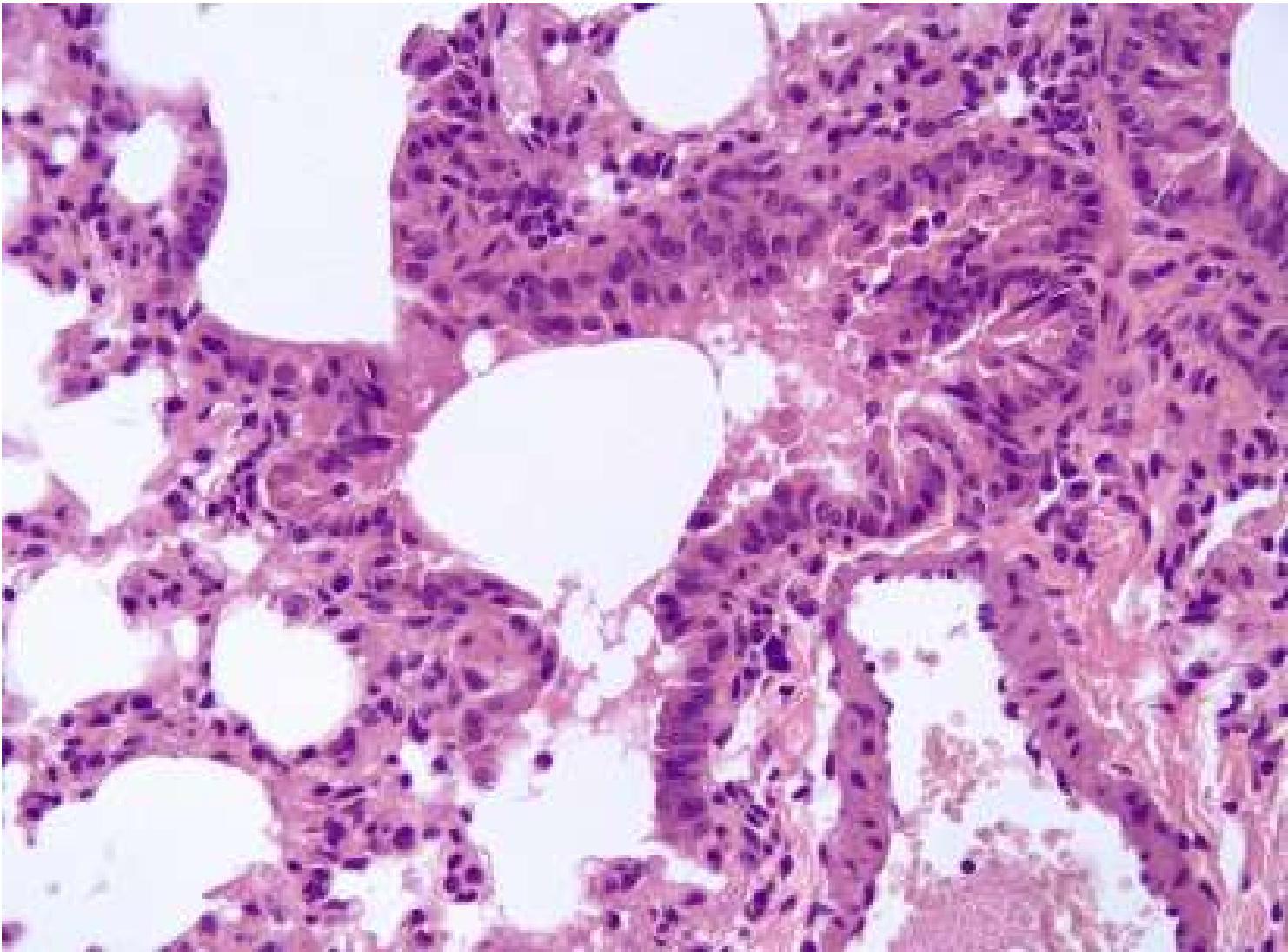

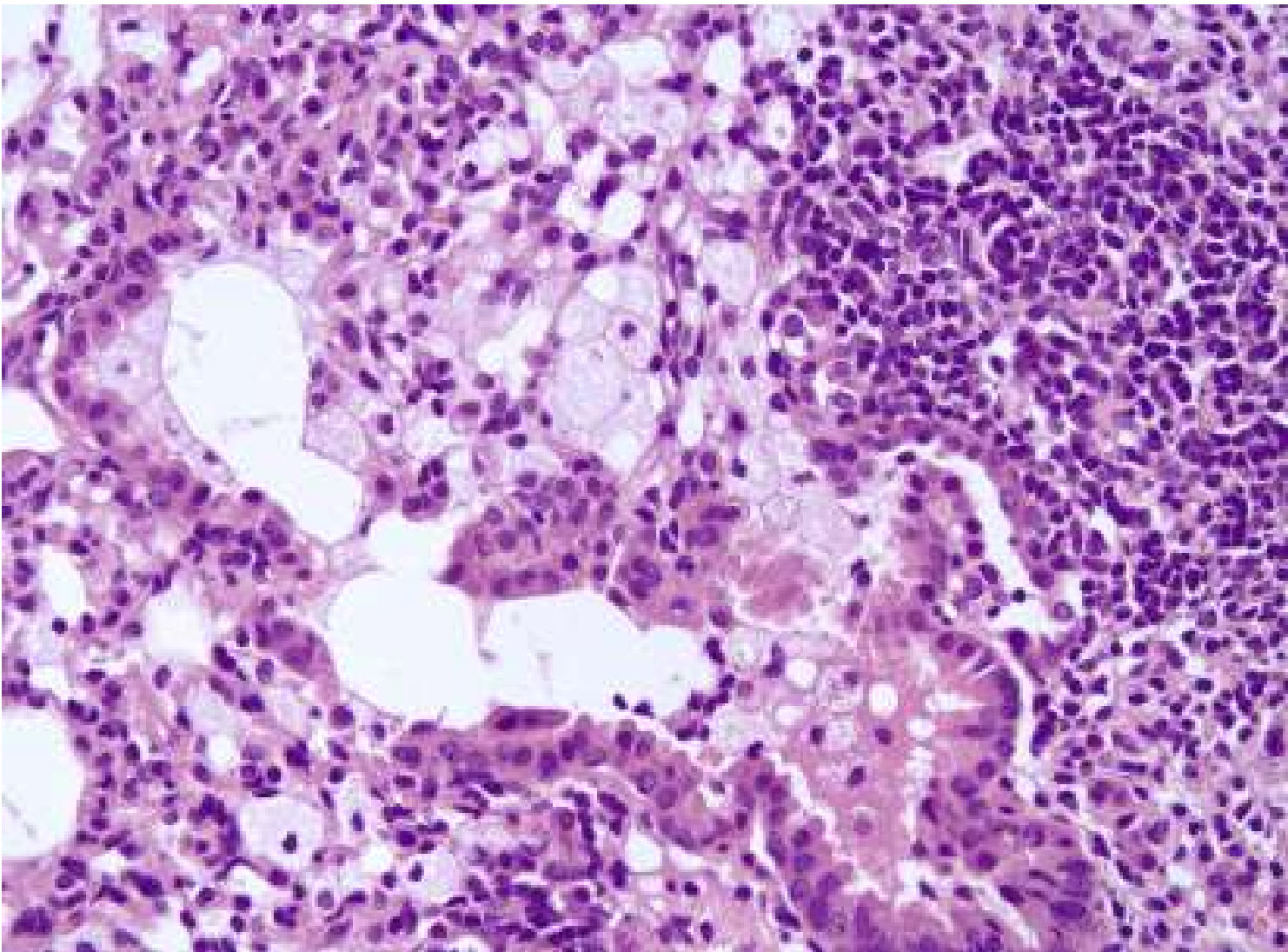

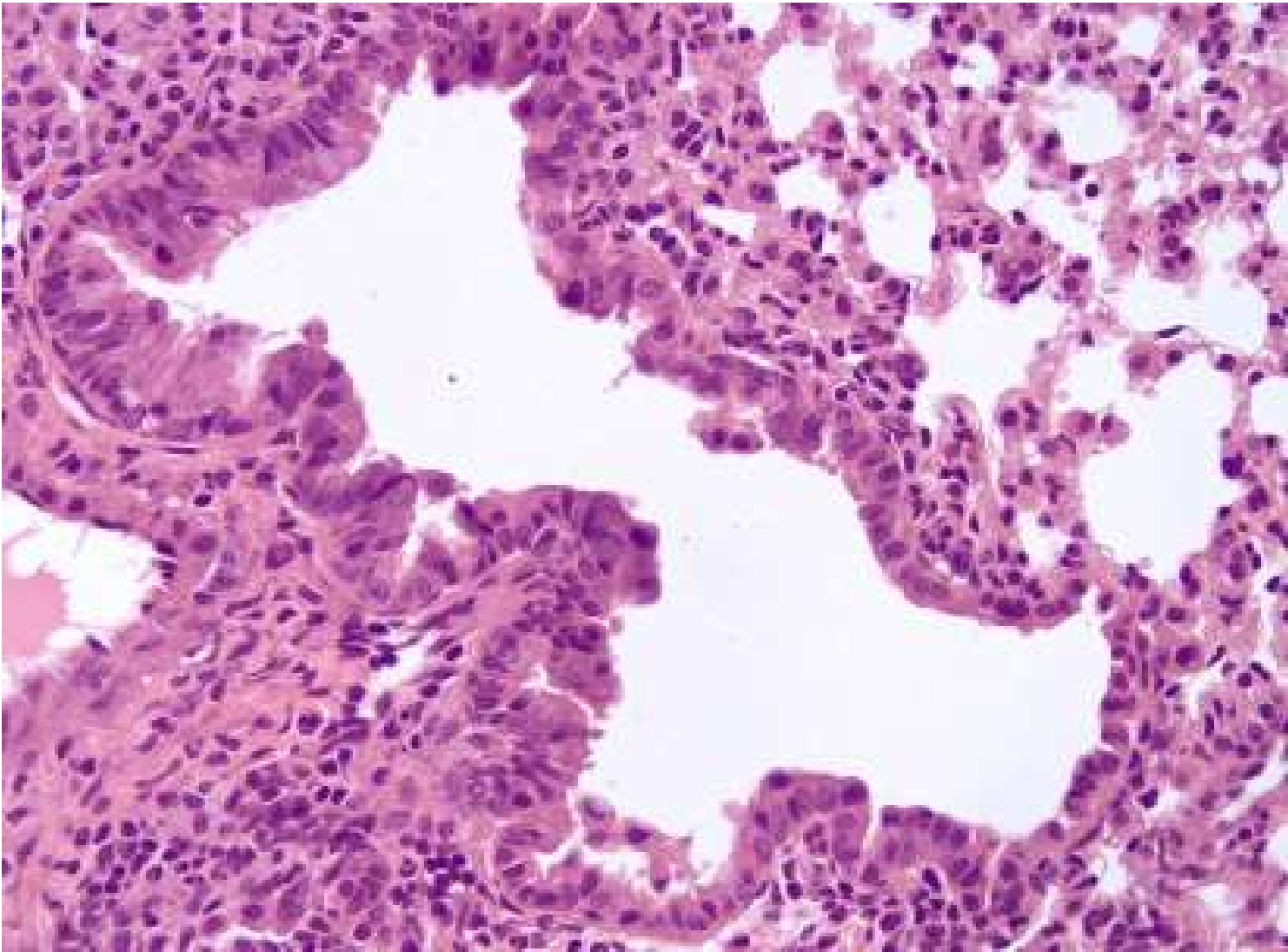

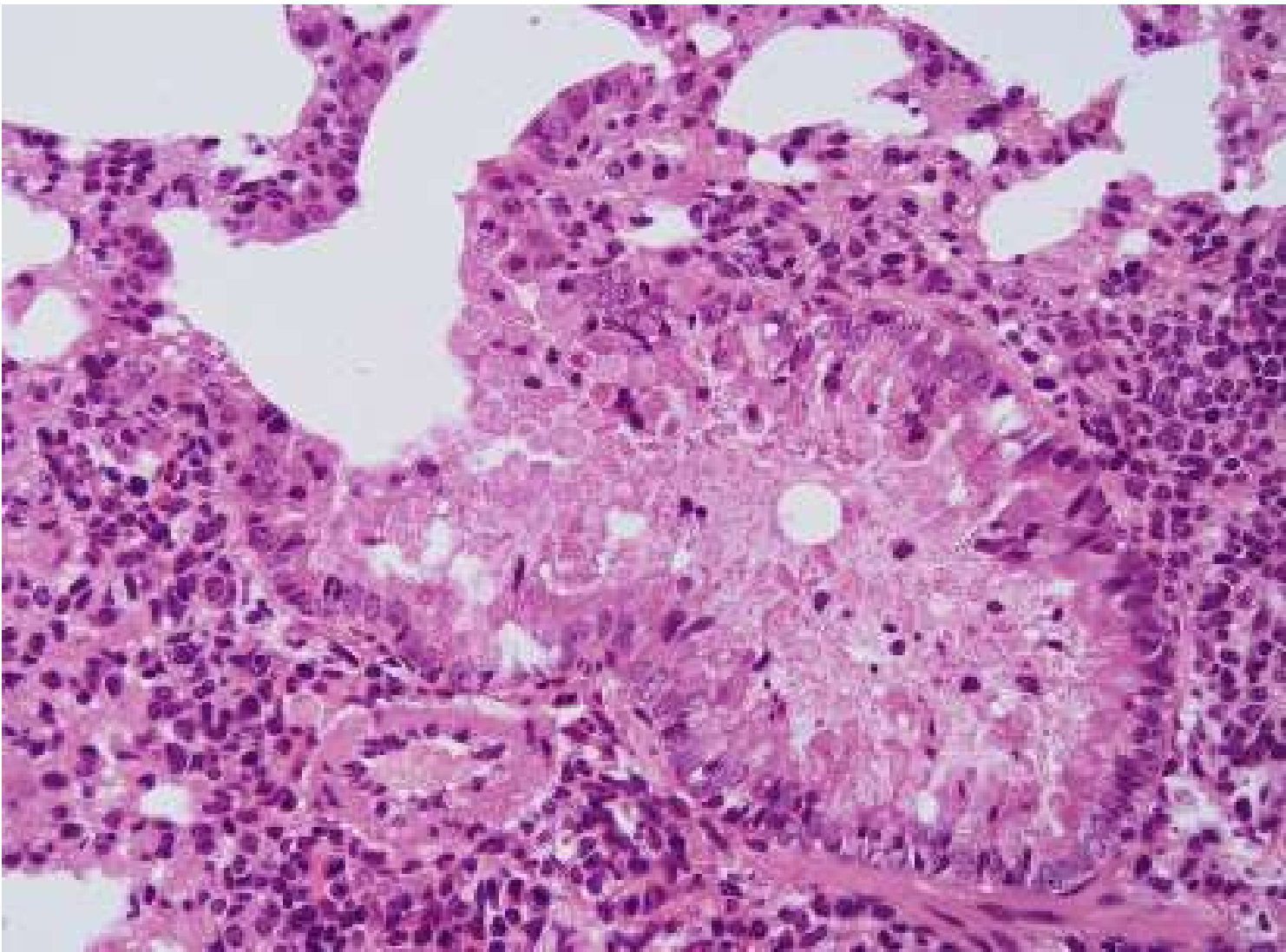

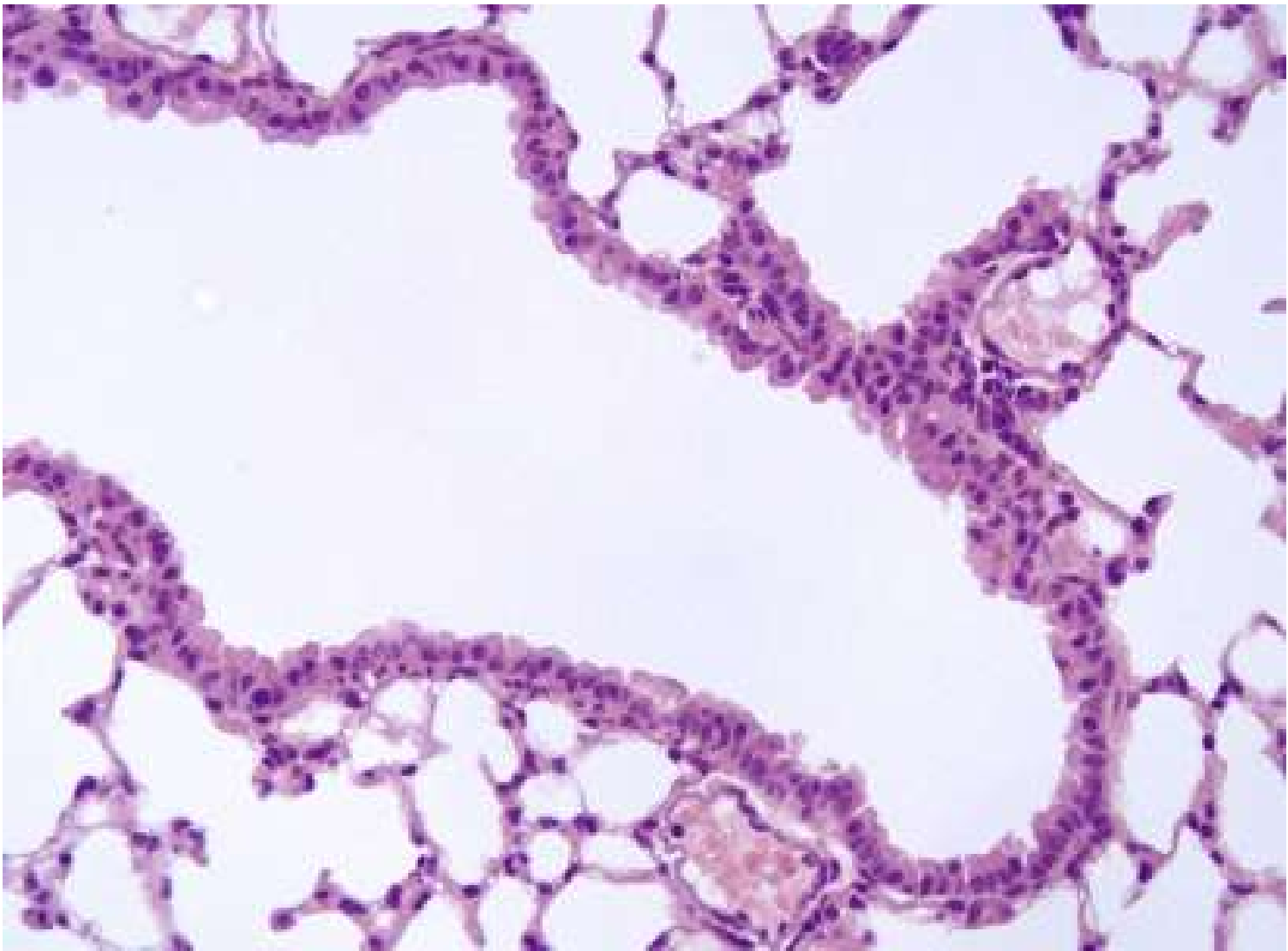

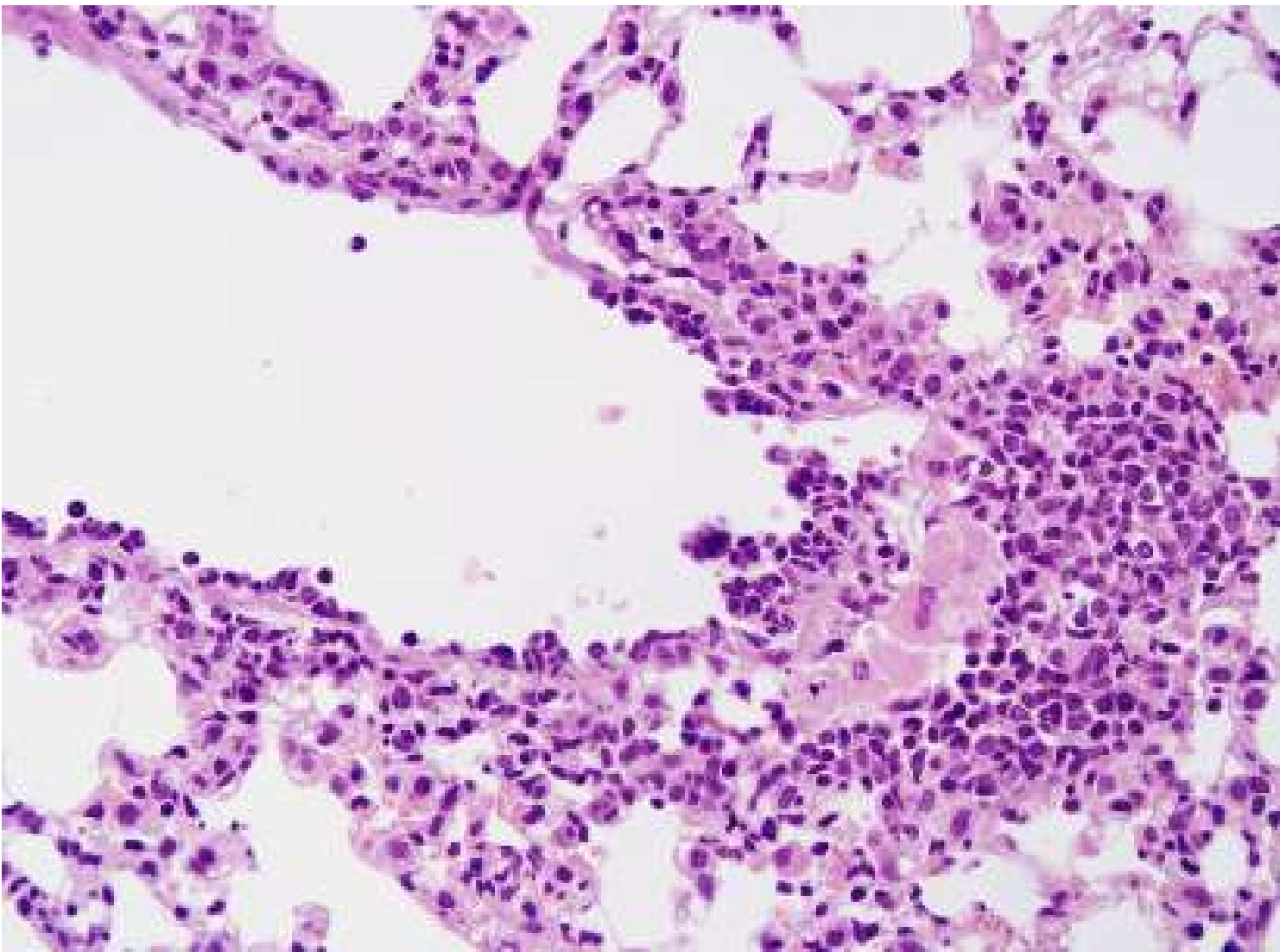

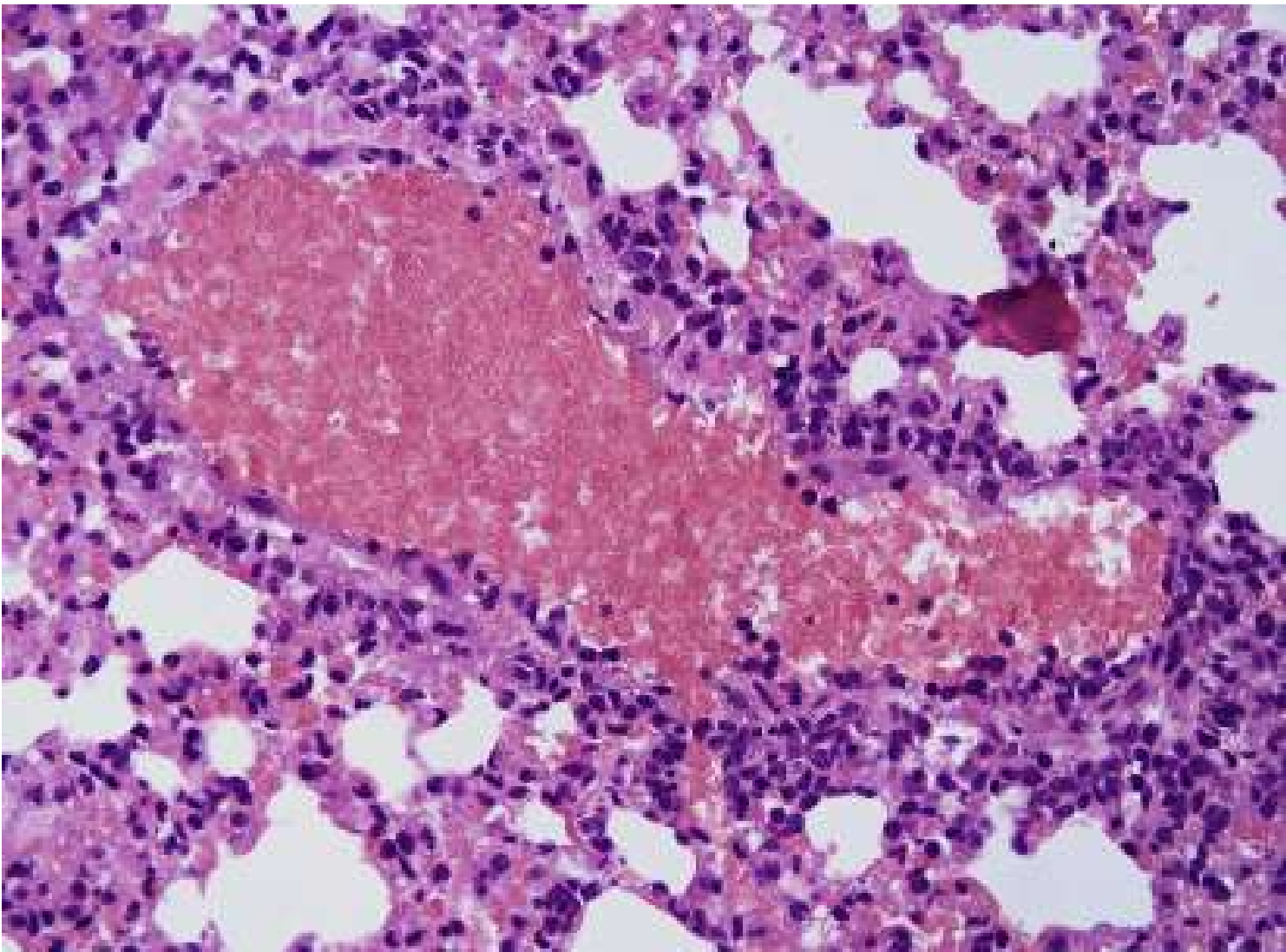

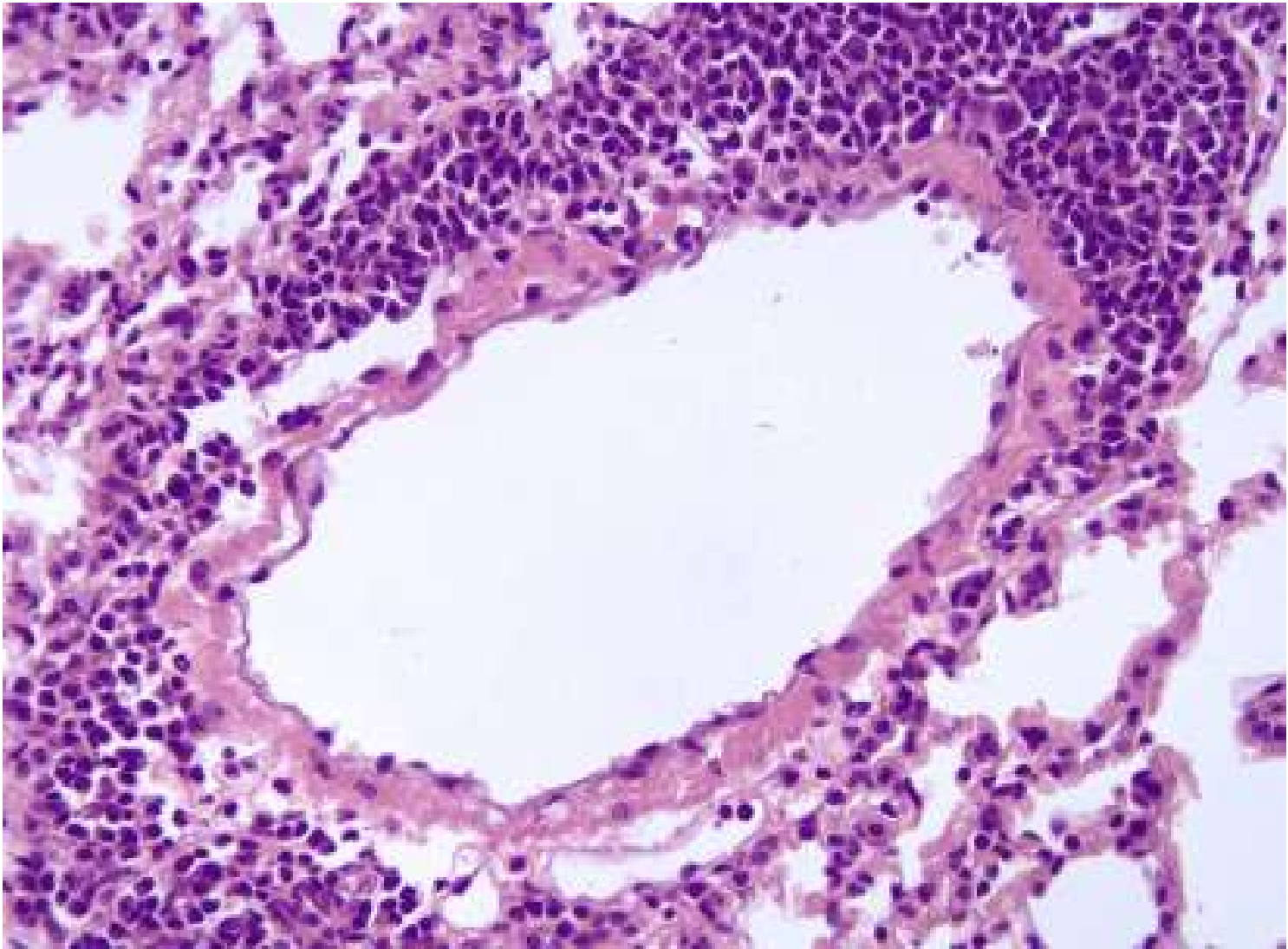

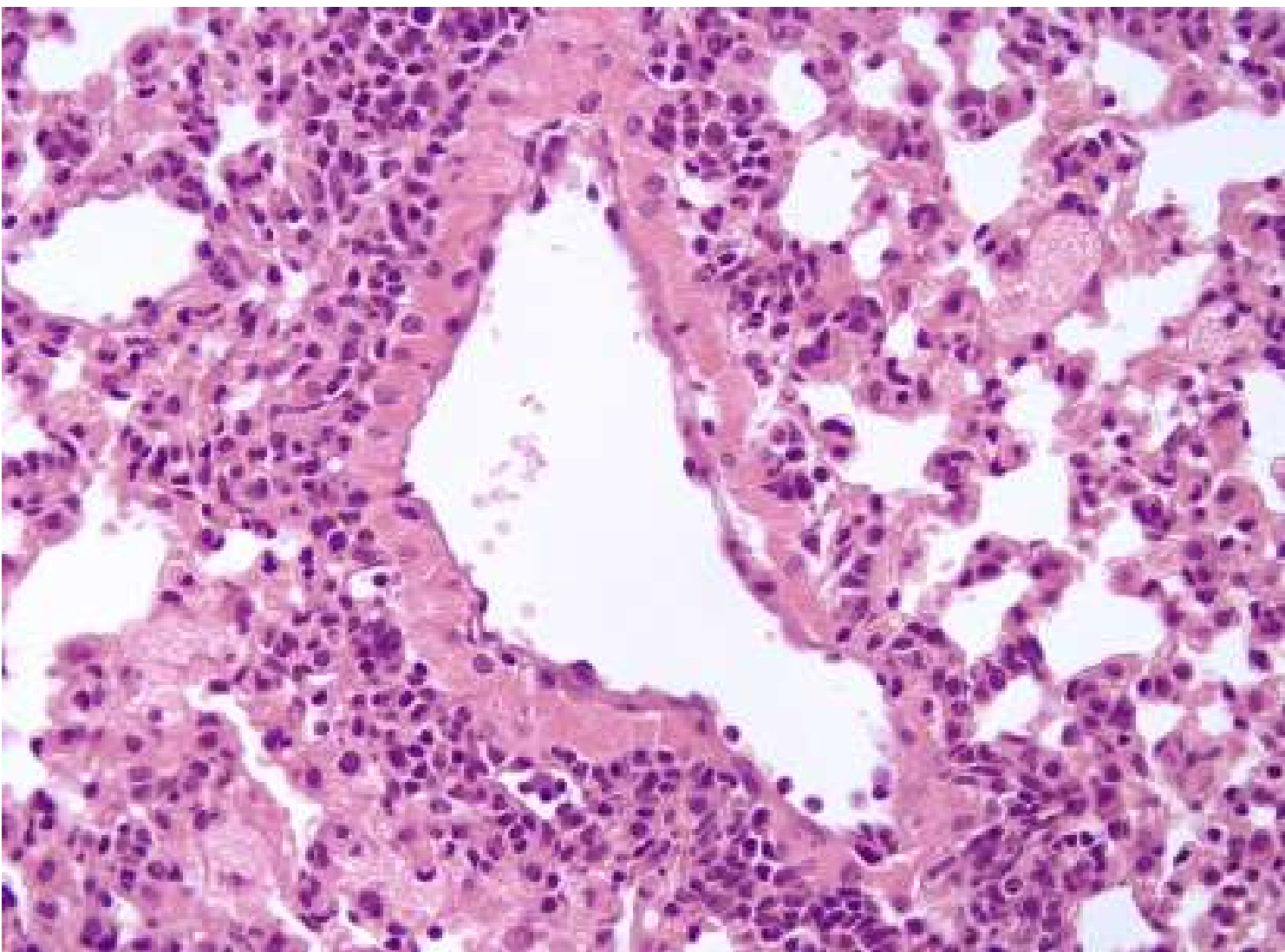

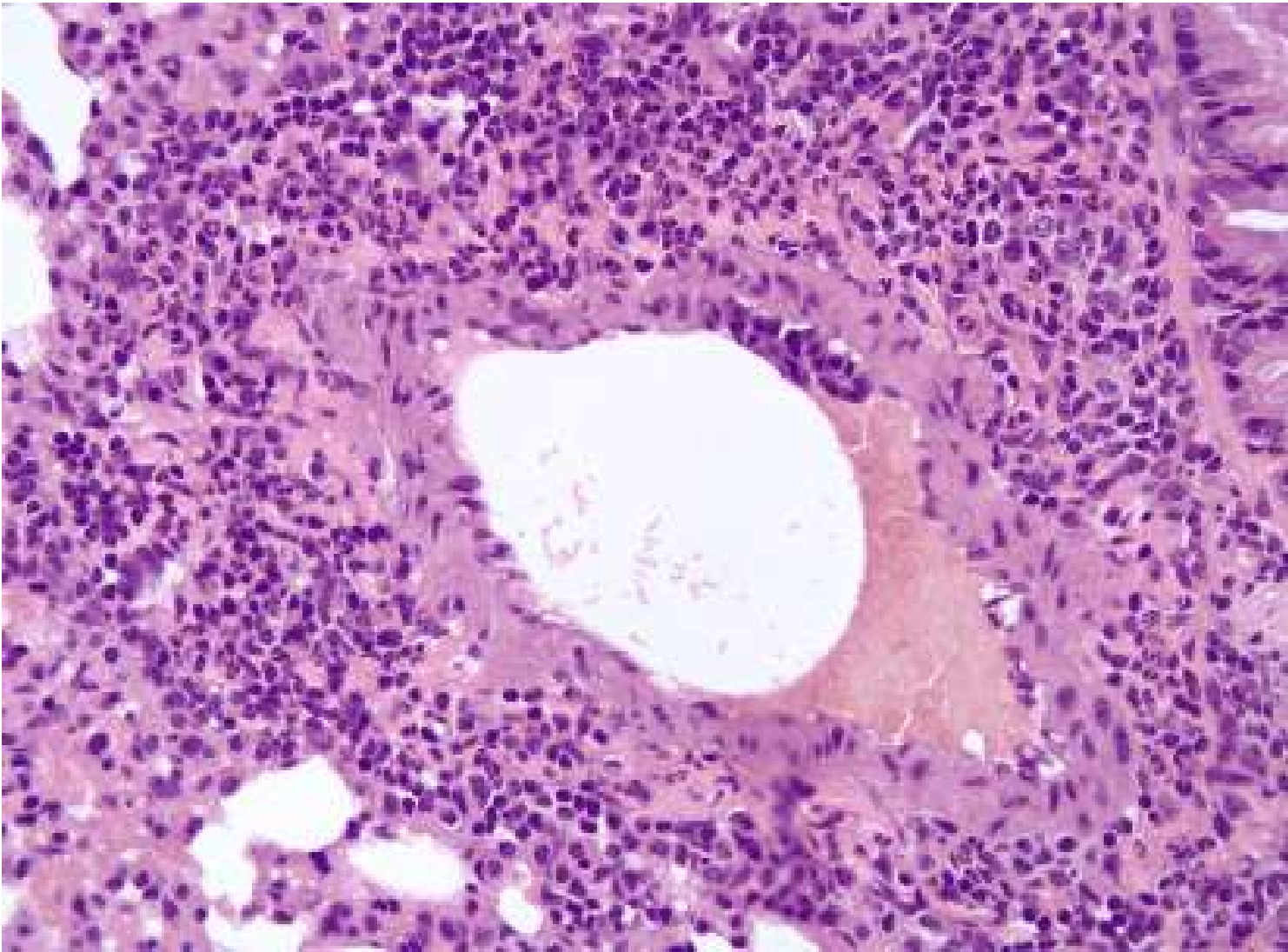

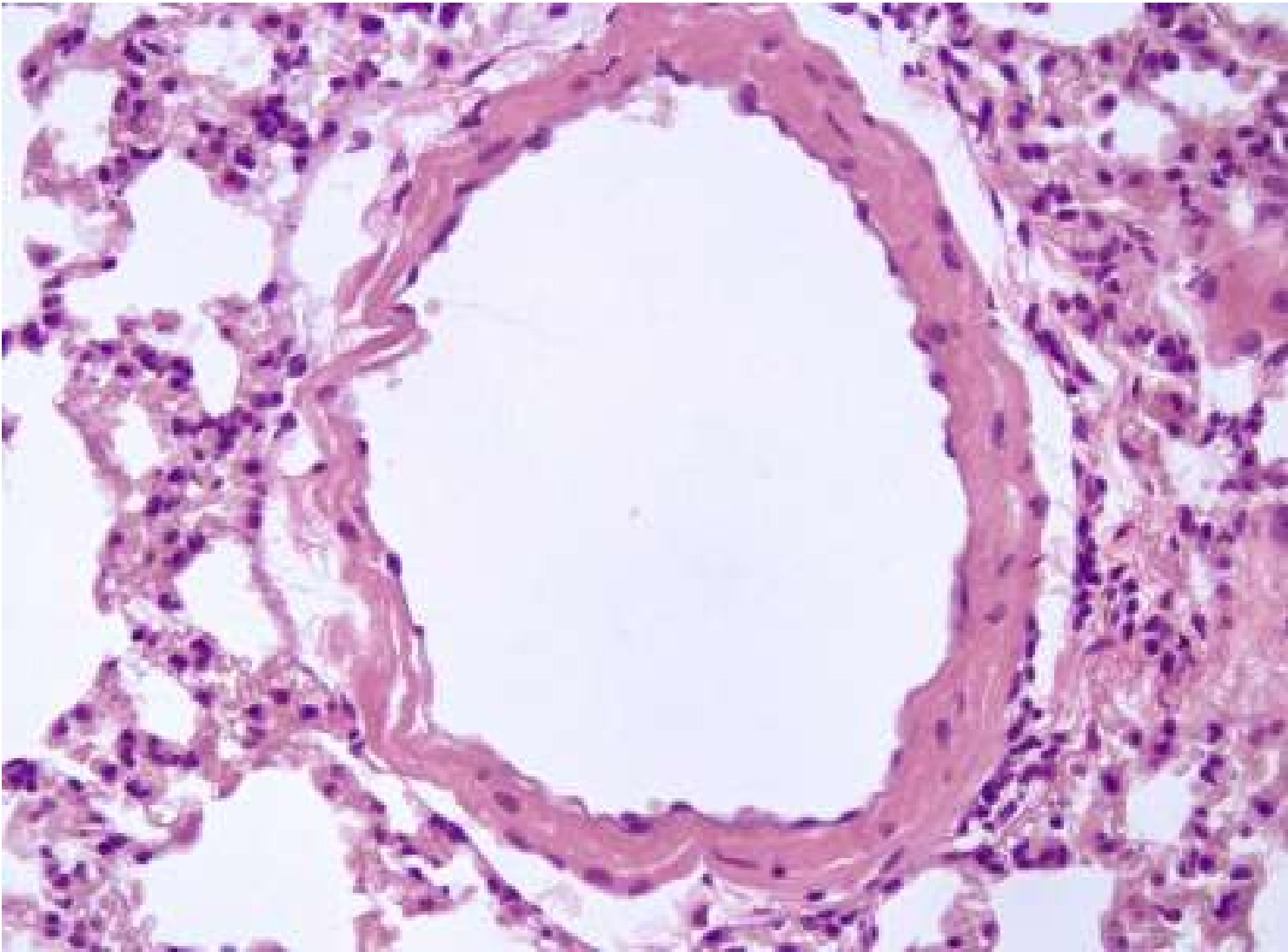

Supplement: Supplementary file 1 [file vaccines-13-00015-s001.zip › original images.pdf]
